# The Influence of Bacterial Inoculants and a Biofertilizer on Maize Cultivation and the Associated Shift in Bacteriobiota During the Growing Season †

**DOI:** 10.3390/plants14121753

**Published:** 2025-06-07

**Authors:** Katarina Kruščić, Aleksandra Jelušić, Matjaž Hladnik, Tamara Janakiev, Jovana Anđelković, Dunja Bandelj, Ivica Dimkić

**Affiliations:** 1 Faculty of Biology, University of Belgrade, Studentski Trg 16, 11158 Belgrade, Serbia; katarina.kruscic@bio.bg.ac.rs (K.K.); tamara.janakiev@bio.bg.ac.rs (T.J.); 2 Department of Life Sciences, Institute for Multidisciplinary Research, University of Belgrade, 11030 Belgrade, Serbia; aleksandra.jelusic@imsi.rs; 3 Faculty of Mathematics, Natural Sciences and Information Technologies (UP FAMNIT), University of Primorska, Glagoljaška 8, Sl-6000 Koper, Slovenia; matjaz.hladnik@famnit.upr.si (M.H.); dunja.bandelj@famnit.upr.si (D.B.); 4 Faculty of Sciences and Mathematics, University of Niš, Višegradska 33, 18106 Niš, Serbia; jovana.andjelkovic@pmf.edu.rs

**Keywords:** maize, plant growth-promoting rhizobacteria (PGPR), poultry manure, microbiome, biocontrol, sustainable agriculture

## Abstract

Maize (*Zea mays* L.) relies heavily on nitrogen and phosphorus inputs, typically supplied through organic and inorganic fertilizers. However, excessive agrochemical use threatens soil fertility and environmental health. Sustainable alternatives, such as poultry manure (PM) and plant growth-promoting rhizobacteria (PGPR), offer promising solutions. This study examines the effects of a phytobiotic bacterial formulation (PHY), composed of *Bacillus subtilis* and *Microbacterium* sp., applied alone and in combination with PM, on maize’s rhizosphere bacteriobiome across key growth stages. Field trials included four treatments: a control, PHY-coated seeds, PM, and combined PHY_PM. The results show that early in development, the PM-treated rhizospheres increased the abundance of beneficial genera such as *Sphingomonas*, *Microvirga*, and *Streptomyces*, though levels declined in later stages. The PHY_PM-treated roots in the seedling phase showed a reduced abundance of taxa like *Chryseobacterium*, *Pedobacter*, *Phyllobacterium*, *Sphingobacterium*, and *Stenotrophomonas*, but this effect did not persist. In the PM-treated roots, *Flavisolibacter* was significantly enriched at harvesting. Overall, beneficial bacteria improved microbial evenness, and the PHY_PM treatment promoted bacterial diversity and maize growth. A genome analysis of the PHY strains revealed plant-beneficial traits, including nutrient mobilization, stress resilience, and biocontrol potential. This study highlights the complementarity of PM and PGPR, showing how their integration reshapes bacteriobiome and correlates with plant parameters in sustainable agriculture.

## 1. Introduction

Maize (*Zea mays* L.) is among the world’s three most important and widely grown cereals, along with wheat (*Triticum aestivum* L.) and rice (*Oryza sativa* L.) [1]. Due to its genetic potential for high yield and fast growth habits, maize is an extremely nutrient-exhaustive crop, requiring nitrogen (N) and phosphorus (P) more than other essential elements for the development of all growth phenophases [2]. Therefore, appropriate amounts of the essential nutrients needed for the enhancement of maize yields and soil fertility are provided through the integration of organic and inorganic fertilizers [2].

The excessive and long-term application of high doses of agrochemicals, like inorganic fertilizers and pesticides to attain high crop yields, has led to adverse consequences for human health and the environment [3]. Frequent applications of such chemicals leads to a decrease in soil fertility and exposes crops to various diseases [4]. Although their indiscriminate use is inevitable to meet the increasing demand of the growing human population for a healthy food supply, organic fertilizers (animal manure, crop residues, compost, green manure, etc.) and biofertilizers (such as microbial fertilizers) are becoming recognized as effective, economically feasible, and environmentally sound alternatives for sustainable agriculture [5,6,7].

Organic fertilizers provide plants with a variety of valuable macro- and micro-nutrients and have the dual advantage of replenishing depleted soil fertility and reducing farming waste [8,9]. However, their effect on crop yields is slow and variable in the short term due to the inconsistency in nutrient content; thus, farmers are reluctant to use them in their cropping system [10]. Due to known fertilizing properties, animal manures are often used on agricultural land. In addition to being an important resource of organic matters, useful microorganisms, and nutrients [primary (N, P, K and S), secondary (Ca and Mg), and micronutrients (B, Cl, Cu, Fe, Mn, Mo and Zn)], animal manures also contain toxic metals and pathogenic microorganisms [11]. The most commonly used sources of animal manures worldwide are cattle and poultry; however, it is not uncommon to use manures from horses, sheep, goats, etc. [12]. The effect of poultry manure on maize yields was previously evaluated in a study conducted by Kolawole [13], suggesting that the studied grain yield was undoubtedly higher after manure treatment compared to the yield without any treatment applied. The mentioned study also highlighted the importance of application time in achieving the desired maize yield, emphasizing that planting is the best moment for the application of poultry manure. Similarly, Adeyemo et al. [14] also confirmed that the use of poultry manure increased maize yield parameters (fresh shoot biomass, dry shoot biomass, cob weight, dry grain weight, wet grain weight, and 1000 grain weight), regardless of differences in soil types.

Numerous microbial taxa, applied alone or in a consortium, are being exploited as biofertilizers due to their potential to competitively colonize the plants’ rhizosphere, rhizoplane, or root interior and to assist nutrient uptake [15,16,17]. Unlike chemical and organic fertilizers, microbial-based inoculants do not directly provide crops with any nutrients. Instead, they are involved in processes like nitrogen fixation, phosphate solubilization, and the promotion of plant growth or may possess a combination of all or some of these properties [18,19]. *Rhizobia* and plant growth-promoting rhizobacteria (PGPR) from the genera *Bacillus*, *Pseudomonas*, *Azotobacter*, *Azospirillum*, *Bradyrhizobium*, *Enterobacter*, *Mezorhizobium*, etc., are nowadays intensively being used for making biofertilizers [20]. While rhizobia fix atmospheric nitrogen gas (N_2_) in plant root nodules, PGPR provides not just a positive impact on soil fertility but also influences the promotion of plant growth by facilitating their tolerance to biotic and abiotic stresses and by supporting their nutrition and disease control using a plethora of mechanisms [19,21]. Depending on their mode of action, PGPR can be separated into three categories, i.e., biopesticides, biofertilizers, and phytostimulators [22]. The efficacy of microbial-based inoculants is influenced by a multitude of factors under field conditions; therefore, the potential candidates for biofertilizers should be selected based on their performance under field conditions, based on a wide range of crops grown across diverse soil types, and under different environmental conditions [23]. Finally, although biofertilizers are recognized as a promising strategy for sustainable intensification of agriculture, the question of their effectiveness, which depends on the formulation, application method, and optimal conditions required for their best activity and which taxa or their combinations provide the best benefits, remains open [24].

This study aimed to evaluate the impact of the microbial inoculant of a bacterial phytobiotic formulation on native maize bacteriobiome during key growth phenophases under field conditions, as well as to compare whether there are differences in efficacy between the phytobiotic formulation, poultry manure, and their combination based on yield parameters.

The selection of *Bacillus subtilis* subsp. *subtilis* and *Microbacterium* sp. for the phytobiotic formulation was based on their previously established complementary plant growth-promoting traits and complementary growth. *B. subtilis* is renowned for its ability to enhance plant growth through multiple mechanisms, including the production of phytohormones (e.g., indole-3-acetic acid), the solubilization of phosphorus, the synthesis of siderophores, and the induction of systemic resistance against pathogens [25,26,27]. Its capacity to form resilient endospores also ensures survival under adverse environmental conditions, making it a reliable inoculant. On the other hand, *Microbacterium* species have been identified as plant growth-promoting bacteria capable of producing phytohormones and enhancing nutrient uptake, particularly phosphorus and nitrogen, thereby supporting plant development under nutrient-limited conditions [28,29]. The co-inoculation of these two species aims to synergistically exploit their mechanisms to promote plant growth and resilience.

We hypothesize that co-application of a phytobiotic formulation with poultry manure will synergistically stimulate beneficial microbial taxa, increase microbial diversity, and positively influence plant performance in the rhizosphere, roots, and seed microbiomes of maize across developmental stages.

## 2. Results

### 2.1. Raw Data Analysis

We examined the roots and rhizosphere soil of maize plants under different treatments across three phenophases (II: seedling; III: flowering; IV: harvesting), as well as seeds in the harvesting phase. The treatments were defined as follows: T1—uninoculated seeds with no poultry manure (“uninoculated”); T2—coated seeds with a consortium of the phytobiotic formulation (PHY) based on two beneficial strains with no PM; T3—uninoculated seeds fertilized with PM (PM); and T4—uninoculated seeds with PM that were additionally treated with PHY (PHY_PM). In total 11,211,159 reads of the amplified V4 16S rRNA region were obtained for 96 samples (including samples of soil, roots, manure, and seeds). After denoising, quality filtering, and taxonomy filtering, normalization with rarefaction was used as stated in the Section 4. A full summary of the sequence read processing for all samples across different treatments and phenophases can be found in Supplementary Table S1.

### 2.2. Alpha Diversity

**Figure 1:**
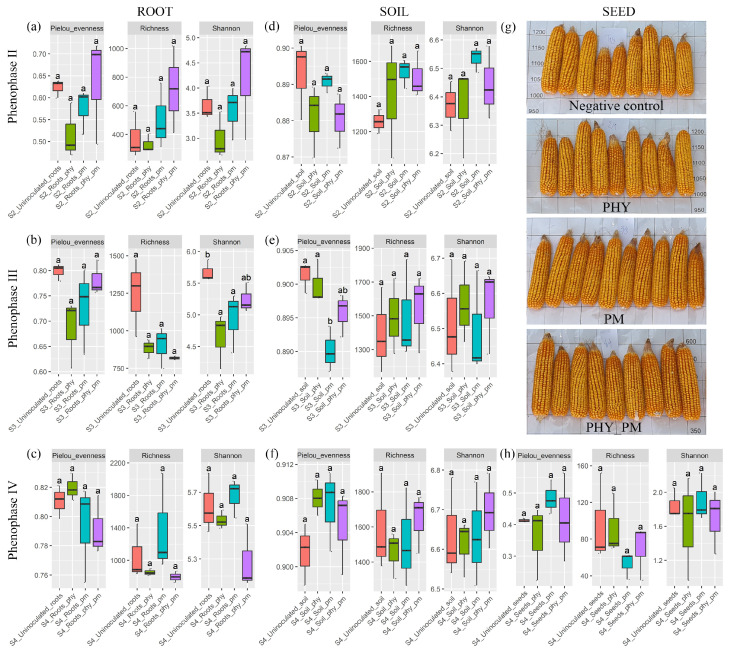
Alpha diversity, represented by Pielou’s evenness and richness and the Shannon index, for root samples (**a**–**c**), soil samples (**d**–**f**), and seed samples (**h**) in phenophases II, III, and IV. Panel (**g**) shows harvested corncobs in phenophase IV. Boxplots were construed based on three replicates of each treatment. According to Tukey’s HSD test, values marked with the same letter within the each boxplot were not statistically significant.

Alpha diversity was assessed using Pielou’s evenness, richness and the Shannon index. For the roots, Pielou’s evenness increased from phenophase II to IV. In phenophases II and III, T4 showed the highest median values of evenness (Figure 1a,b), while in phenophase IV, T2 was highest (Figure 1c). Similarly, richness increased across phenophases; however, T4 had the highest median richness in phenophase II, T1 in phenophase III (Figure 1a,b), and T3 in phenophase IV (Figure 1c). The Shannon index for roots followed the same pattern as richness. Notably, the only statistically significant difference, based on the Kruskal–Wallis test (p.adj < 0.05), was observed in phenophase III for the root Shannon index, where a negative control (T1) had a significantly higher value compared to T2 and T3 (p.adj = 0.038) (Figure 1b). Moreover, in phenophases II and III, T2 consistently recorded the lowest median values for all three indexes in roots (Figure 1a,b), whereas in phenophase IV, the diversity indexes for T2 increased, and T4 then exhibited the lowest median values (Figure 1c). For rhizosphere soil, Pielou’s evenness also increased from phenophase II to IV. In phenophases II and III, T4 showed the highest median value of evenness (Figure 1d,e), while in phenophase IV, T3 had the highest value (Figure 1f). The only statistically significant difference, according to the Kruskal–Wallis test, was observed in phenophase III, where soil samples from T1 and T2 that were grouped together were significantly different from those of T3 (p.adj = 0.048) (Figure 1e). Although rhizosphere richness increased through the phenophases—with T2 having the highest richness in phenophase II (Figure 1d) and T4 in the later stages—no statistically significant differences were found among the treatments. For the rhizosphere’s Shannon index, T3 had the highest median in phenophase II (Figure 1d), while T4 showed the highest values in phenophases III (Figure 1e) and IV (Figure 1f); however, these differences were not statistically significant.

In the harvested seeds from the fourth (IV) phenophase, the highest median value for richness and the Shannon index was observed in T4, while Pielou’s evenness was highest in T3 (Figure 1h).

### 2.3. Beta Diversity

The time of sampling significantly explained approximately 60.5% of the variance in root microbial community structure (PERMANOVA, *p* = 0.001), while treatments accounted for an additional, smaller but still significant 4.4% (*p* = 0.001). However, pairwise comparisons of individual phenophases did not lead to statistically significant differences, likely due to limited replications within each treatment. Similar results were obtained for rhizosphere samples, although less variability was explained by the different sampling points (34.6%) and treatments (6.3%). Both factors had significant influence (*p* = 0.001), but a pairwise analysis did not reveal significant differences. No significant differences were found for seeds after harvesting.

**Figure 2:**
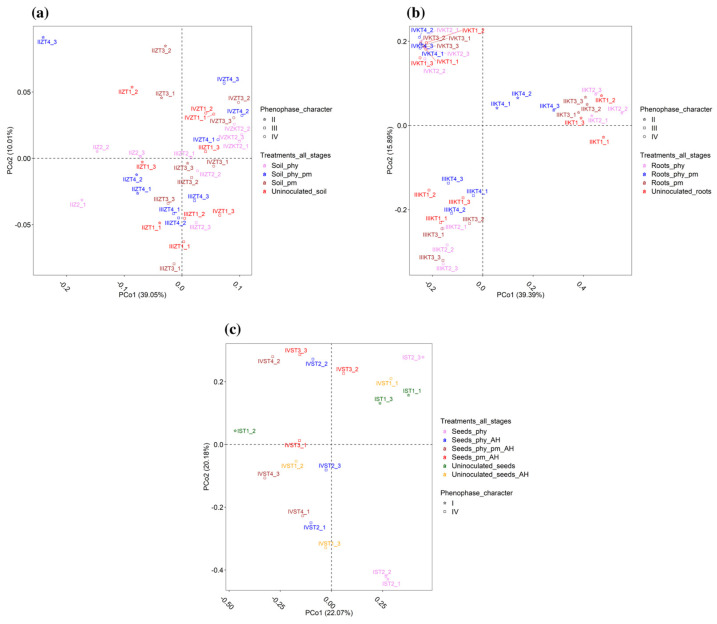
Principal Coordinate Analysis (PCoA) illustrating genus-level differences among the 36 analyzed maize samples from the rhizosphere (**a**) and root (**b**), treated with FitoBiotic Basic (_PHY)—T2, poultry manure (_PM)—T3, their combination (_PHY_PM)—T4, and uninoculated controls—T1. Samples were observed across three phenophases: II—seedlings, III—flowering, and IV—harvesting. Panel (**c**) shows genus-level differences in 18 maize seed samples collected from plants treated with FitoBiotic Basic (seeds_PHY), poultry manure (seeds_PM), their combination (seeds_PHY_PM), and uninoculated controls (seeds_control). These were analyzed at two phenophases: I—pre-harvesting and IV—post-harvesting (AH).

The PCoA analysis presented in Figure 2a shows the beta diversity of the maize rhizosphere samples, including negative controls and treatments, across four phenophases (I–IV) at the genus level. The first principal coordinate (PCo1), explaining 39.05% of the variance, reveals a close association among all soil samples from phenophase IV, including IV_uninoculated soil_1/_2/_3, IV_soil_PHY_1/_2/_3, IV_soil_PM_1/_2/_3, and IV_soil_PHY_PM_1/_2/_3.

Additionally, certain samples from phenophases II and III—specifically II_soil_PM_3 from phenophase II and III_uninoculated soil_1/_2/_3, III_soil_PHY_1/_2/_3, III_soil_PM_2, and III_soil_PHY_PM_3 from phenophase III—also showed similarities to this group. On the other hand, the remaining soil samples from phenophase II, including II_uninoculated soil_1/_2/_3, II_soil_PHY_1/_2/_3, II_soil_PM_1/_2, and II_soil_PHY_PM_1/_2/_3, along with some samples from phenophase III, such as III_soil_PM_1/_3 and III_soil_PHY_PM_1/_2, formed a separate cluster, indicating a more distinct microbial composition compared to the previously mentioned samples. The second principal coordinate (PCo2), which accounts for 10.01% of the variance, further distinguishes the samples. A group of 18 samples showed a higher degree of similarity, including IV_uninoculated soil_1/_2, IV_soil_PHY_1/_2/_3, IV_soil_PM_2/_3, and IV_soil_PHY_PM_1/_2/_3 from phenophase IV, along with II_uninoculated soil_2, II_soil_PHY_2/_3, II_soil_PM_1/_2, II_soil_PHY_PM_3, III_uninoculated soil_3, and III_soil_PHY_1 from earlier phenophases. In contrast, another distinct group of 18 samples included II_uninoculated soil_1/_3, II_soil_PHY_1, II_soil_PM_3, II_soil_PHY_PM_1/_2, III_uninoculated soil_1/_2, III_soil_PHY_2/_3, III_soil_PM_1/_2/_3, III_soil_PHY_PM_1/_2/_3, IV_uninoculated soil_3, and IV_soil_PM_1.

The results of a PCoA analysis observed at the genus level, showing differences in the bacterial diversity between the tested roots samples, during three tested phenophases (II–IV) are presented in Figure 2b. According to the PCoA, the first dimension (PCo1 at 39.39%) separated all 12 samples (negative controls and treatments) from phenophase II (II_uninoculated root_1/_2_3, II_ root_PHY_1/_2_3, II_root_PM_1/_2_3, and II_ root_PHY_PM_1/_2_3) from the samples collected in phenophases III (III_uninoculated root_1/_2_3, III_ root_PHY_1/_2_3, III_root_PM_1/_2_3, and III_ root_PHY_PM_1/_2_3) and IV (IV_uninoculated root_1/_2_3, IV_root_PHY_1/_2_3, IV_root_PM_1/_2_3, and IV_ root_PHY_PM_1/_2_3), indicating their greater similarity. According to the second dimension (PCo2 at 15.89%), overall, there is a clear distinction between three phenophases. The phenophase III and IV samples are distinctly separated, indicating that while they may share similarities along PCo1, there are significant differences in bacterial composition that become visible along the second principal coordinate.

Figure 2c presents the results of a PCoA analysis, showing differences between the tested maize seed samples in negative controls (uninoculated seeds pre- and after harvesting) and treatments (PHY, PM, and their combination) at the genus level. Based on the obtained results, differences were observed between treatments and among replicates of the same treatments. A strict separation between the two tested phenophases and/or treatments/negative controls was not observed. According to the first dimension (PCo1 at 22.07%), 11 samples, representing negative controls pre- (I_uninoculated seeds_PH_2) and after (IV_uninoculated seeds_AH_2/_3) harvesting and treatments from phenophase IV, PHY (IV_seeds_PHY_AH_1/_2/_3), PM (IV_seeds_PM_AH_1/_3), and their combination (IV_seeds_PHY_PM_AH_1/_2/_3), were more similar in comparison to the remaining seven samples, i.e., I_uninoculated seeds_PH_1/_3, I_seeds_PHY_1/_2/_3, IV_uninoculated seeds_AH_1, and IV_seeds_PM_AH_2. However, based on the second dimension (PCo2 at 20.18%), the negative control (I_uninoculated seeds_1/_2/_3) and the seed samples treated with PHY (I_seeds_PHY_3), sampled pre-harvesting, as well as the negative control (IV_uninoculated seeds_AH_1) and treatments with PHY (IV_seeds_PHY_AH_2), PM (IV_seeds_PM_AH_2/_3), and their combination (IV_seeds_PHY_PM_AH_2), sampled after harvesting, were more similar among themselves in comparison to the remaining nine seed samples.

### 2.4. Taxonomy

**Figure 3:**
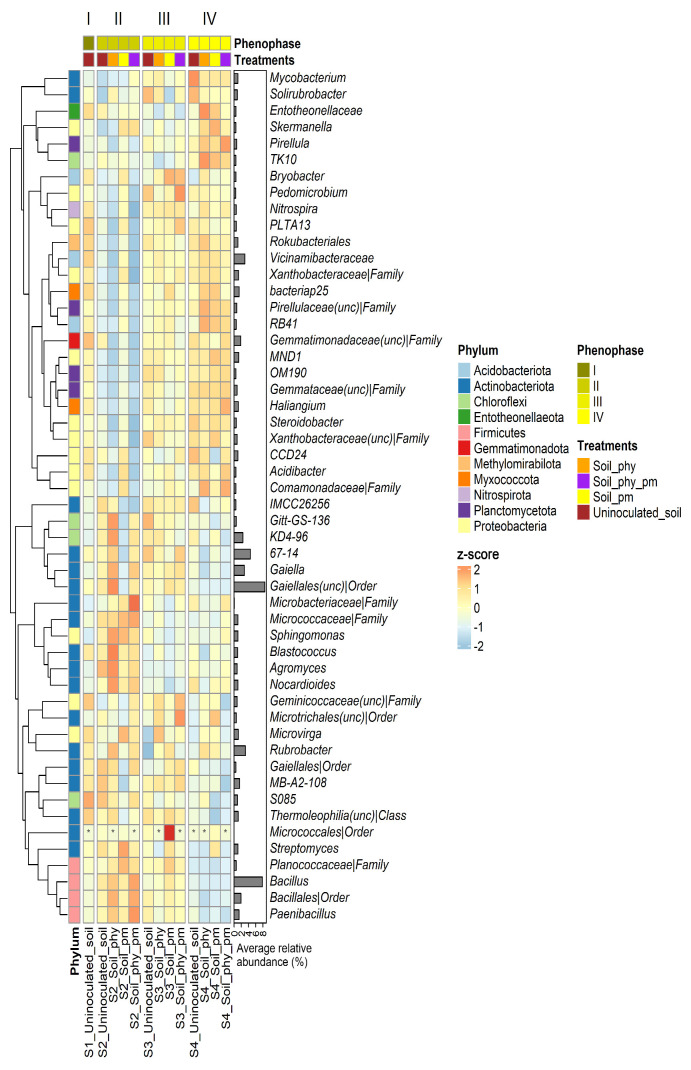
Heatmap of scaled average abundances of the 50 most prevalent taxa across all soil samples, including negative controls and treatments, throughout the four phenophases. The list of taxa was screened for *Microbacterium*, *Microbacterium*, *Micrococcales*, and *Microbacteriaceae*, and these taxa were included if present. An asterisk (*) indicates a zero value.

Data on the effects of the applied treatments (PHY, PM, and their combination) on changes in the composition of the indigenous microbial communities of the maize rhizosphere soil during the tested phenophases (I–IV) are presented in Figure 3. As was generally observed, rhizosphere bacterial communities mostly remained unchanged, regardless of the treatment applied or the tested phenophase. The most abundant genera in all 39 tested soil samples (negative control and treatments) were uncultured *Gaiellales* [7.42% (II_soil_PM)—10.56% (II_soil_PHY)] and *Bacillus* [5.75% (IV_soil_PHY)—11.15% (II_soil_PHY_PM)]. Additionally, high relative abundances of taxa 67-14 (3.79–5.11%), *Vicinamibacteraceae* (1.83–3.57%), *Rubrobacter* (2.50–3.61%), Gaiella (2.35–3.41%), KD4-96 (2.19–2.82%), uncultured *Gemmatimonadaceae* (1.38–2.14%), and *Bacillales* (1.08–2.88%) were observed in the majority of the samples. The negative control from phenophase II (II_uninoculated soil) stood out from the rest of the tested samples (0.15–0.41%) due to the higher relative abundance of *Pseudomonas* (1.72%), while the combined treatment (PHY_PM from the same phenophase II (II_soil_PHY_PM) showed a higher abundance of *Paenibacillus* (2.10%) in comparison to the rest of the samples (0.80–1.85%).

In addition to the heatmap presented at the genus level, Figure S1 in the Supplementary Materials displays a corresponding heatmap of scaled average abundances of the 37 most prevalent bacterial phyla across all soil samples, including both negative controls and treatments, throughout the four analyzed phenophases.

**Figure 4:**
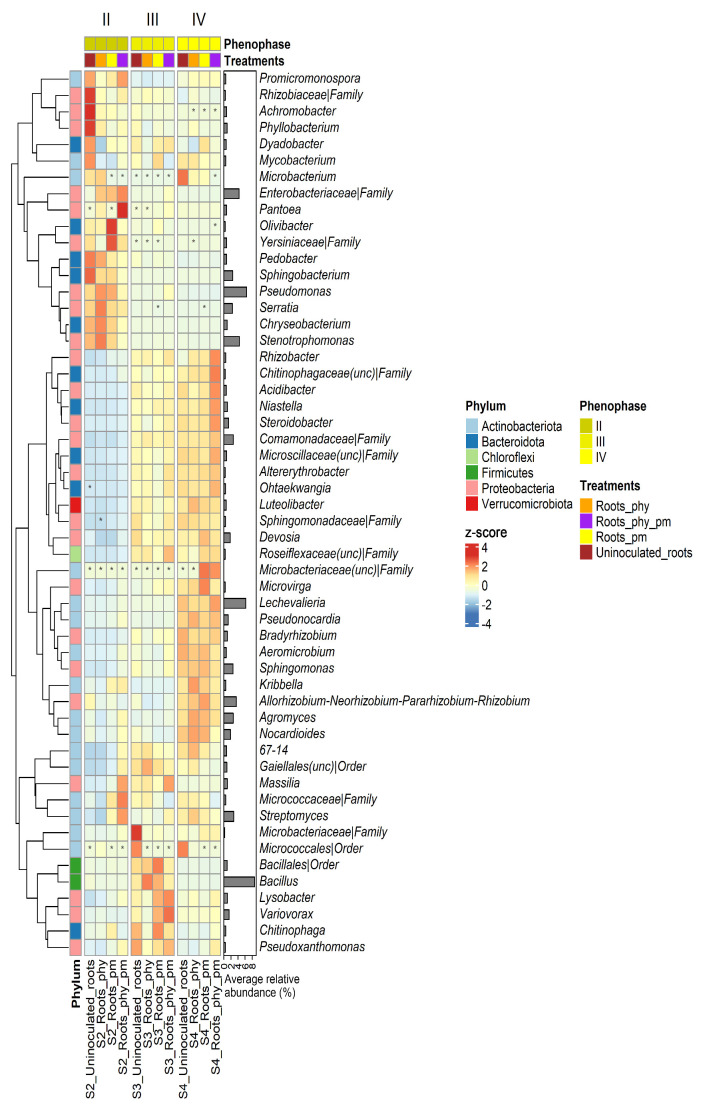
Heatmap of scaled average abundances of the 50 most prevalent taxa across all root samples, including negative controls and treatments, throughout the three phenophases. The list of taxa was screened for *Microbacterium*, *Microbacterium*, *Micrococcales*, and *Microbacteriaceae*, and these taxa were included if present. An asterisk (*) indicates a zero value.

Contrary to the rhizosphere samples, more expressed differences in distribution and relative abundance of different taxa were observed within the tested root samples, both between phenophases and between treatments (Figure 4). The most abundant taxa in the negative control from phenophase II (II_uninoculated root) were *Stenotrophomonas* (14.39%), *Pseudomonas* (13.89%), *Sphingobacterium* (13.34%), *Serratia* (6.54%), and *Achromobacter* (6.33%). Additionally, even though slightly less represented, the taxa *Allorhizobium*-*Neorhizobium*-*Pararhizobium*-*Rhizobium* (3.90%), *Bacillus* (3.81%), *Chryseobacterium* (3.23%), *Phyllobacterium* (2.99%), *Pedobacter* (2.59%), and *Yersiniaceae* (2.23%) were also highly abundant in this sample. After the application of the treatments (PHY, PM, and PHY_PM), only minor differences were observed in the relative abundance of the detected taxa, compared to the negative control. However, the most significant difference that emerged after the treatments’ application in phenophase II was a higher abundance of *Enterobacteriaceae* [13.01% (II_root_PHY), 13.84% (II_root_PM), and 18.21% (II_root_PHY_PM)], especially in the treatment PHY_PM where this taxon was prevalent. Next to the prevalence in *Enterobacteriaceae*, roots treated with PHY_PM differed from the rest of the samples (negative control and treatments) due to the pronounced presence of *Pantoea* (6.61%), Streptomyces (5.02%), *Lechevalieria* (3.41%), *Agromyces* (2.89%), *Massilia* (1.96%), *Nocardioides* (1.81%), and *Sphingomonas* (1.64%). Also, the abundance of *Achromobacter* [0.26% (II_root_PHY_PM)—1.21% (II_root_PHY)] and *Sphingobacterium* [1.88% (II_root_PHY_PM)—7.30% (II_root_PM)] was greatly reduced in all treatments in comparison to the negative control. Additionally, in roots treated with PM, the relative abundance of *Yersiniaceae* (4.84%) was greater in comparison to the other two treatments [0.06% (II_root_PHY)—2.15% (II_root_PHY_PM)]. In the remaining two phenophases [flowering (III) and harvesting (IV)], a complete shift in bacterial community composition occurred in comparison to the seedling phenophase (II). In the flowering phenophase, *Bacillus* was a prevalent taxon in both the negative control (16.45%) and treatments [14.05% (III_root_PHY_PM), 27.98% (III_root_PM), and 34.55% (III_root_PHY)]. Besides *Bacillus*, among the highly abundant taxa in this phenophase were *Pseudomonas* [1.99% (III_root_PHY)—6.78% (III_root_PHY_PM)], *Variovorax* [0.77% (III_root_PHY)—4.85% (III_root_PHY_PM)], *Comamonadaceae* [2.95% (III_uninoculated root)—3.60% (III_root_PHY)], *Bacillales* [1.46% (III_root_PHY_PM)—3.48% (III_root_PM)], *Allorhizobium*-*Neorhizobium*-*Pararhizobium*-*Rhizobium* [1.88% (III_root_PHY)—3.06% (III_uninoculated root)], *Streptomyces* [1.94% (III_root_PM)—3.17% (III_root_PHY_PM)], *Devosia* [1.73% (III_root_PHY)—2.72% (III_uninoculated root)], *Lechevalieria* [2.73% (III_root_PHY_PM)—3.06% (III_root_PHY)], *Sphingomonas* [1.64% (III_root_PM)—2.82% (III_root_PHY_PM)], and *Massilia* [0.96% (III_root_PM)—1.98% (III_root_PHY_PM)], depending on the sample.

Roots treated with the combination of PHY and PM (PHY_PM) stood out due to the higher presence of *Enterobacteriaceae* (3.76%) and *Steroidobacter* (1.88%) in comparison to the other samples from this phenophase where relative abundance of *Enterobacteriaceae* ranged from 0.48% (III_root_PM) to 0.58% (III_uninoculated root) and of *Steroidobacter* from 0.70% (III_root_PHY) to 1.48% (III_uninoculated root). In the harvesting of phenophase (IV), a new shift in the dominant bacterial communities occurred in comparison to phenophases II and III. However, relative abundance of the most significant genera did not vary much between treatments within this phenophase. Genus *Lechevalieria* [12.12% (IV_root_PM)—16.89% (IV_root_PHY_PM)] prevailed in this phenophase, both in negative control and treatments, followed by *Allorhizobium-Neorhizobium-Pararhizobium-Rhizobium* [4.82% IV_root_PHY_PM)—6.28% (IV_root_PM)], *Agromyces* [3.70% (IV_uninoculated root)—4.70% (IV_root_PHY)], *Nocardioides* [1.98% (IV_root_PHY_PM)—4.25% (IV_root_PHY)], *Streptomyces* [2.61% (IV_root_PHY_PM)—4.31% (IV_root_PHY)], *Comamonadaceae* [3.76% (IV_root_PHY)—4.82% (IV_root_PHY_PM)], *Sphingomonas* [4.02% (IV_root_PHY_PM)—5.11% (IV_root_PM)], *Devosia* [2.17% (IV_uninoculated root)—3.05% (IV_root_PM)], *Steroidobacter* [2.14% (IV_root_PHY)—3.49% (IV_root_PHY_PM), *Pseudonocardia* [2.46% (IV_root_PM)—2.99% (IV_root_PHY)], and *Bradyrhizobium* [1.73% (IV_root_PHY)—2.21% (IV_uninoculated root)].

Figure S2 in the Supplementary Materials displays a corresponding heatmap of scaled average abundances of the 34 most prevalent bacterial phyla across all root samples, including both negative controls and treatments, throughout the three analyzed phenophases.

**Figure 5:**
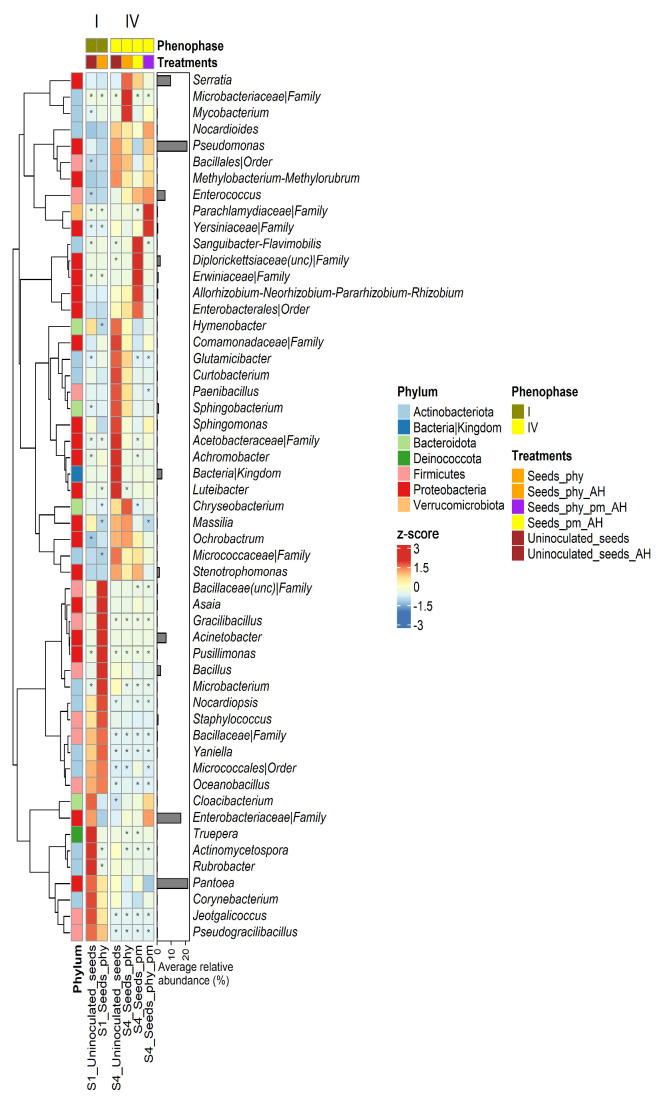
Heatmap of scaled average abundances of the 50 most prevalent taxa across all seed samples, including negative controls and treatments, before and after harvesting. The list of taxa was screened for *Microbacterium*, *Microbacterium*, *Micrococcales*, and *Microbacteriaceae*, and these taxa were included if present. An asterisk (*) indicates a zero value.

Figure 5 shows the distribution and abundance of the taxa detected in seeds during phenophases I (uninoculated and inoculated seeds pre-harvesting) and IV (uninoculated and treated seeds after harvesting). Lower bacterial diversity was observed in the seeds compared to the soil, root, and manure. Also, a reduction in the overall bacterial diversity was observed in phenophase IV in comparison to phenophase I.

Among the detected taxa, the most abundant ones in the uninoculated seeds pre-harvesting (I_uninoculated seeds) were *Pantoea* (40.11%), *Enterobacteriaceae* (34.61%), and *Pseudomonas* (15.45%). However, in the seeds treated with PHY (I_seeds_PHY), the most abundant genus was *Acinetobacter* (36.96%), followed by *Pantoea* (27.275%), *Pseudomonas* (12.30%), and *Bacillus* (10.77%). Differences in the relative abundance of the detected taxa in the seed samples after harvesting were observed between negative controls and treatments. Like in phenophase I, among the most abundant genera in phenophase IV were *Pantoea* [8.59% (IV_seeds_PHY_PM)—21.05% (IV_uninoculates seeds)] and *Pseudomonas* [11.91% (IV_seeds_PM)—32.00% (IV_uninoculates seeds)]. *Enterobacteriaceae* stood out as the most abundant taxa in sample IV_seeds_PHY_PM (34.45%), while it was less represented [6.61% (IV_uninoculates seeds)—13.51% (IV_seeds_PHY)] in the rest of the samples from this phenophase. A high relative abundance of unidentified bacterial taxa (19.13%) was characteristic only for the negative control (IV_uninoculates seeds), while its abundance in the treatments ranged from 0.04% (IV_seeds_PHY) to 0.86% (IV_seeds_PM). Conversely, a high abundance of *Serratia* [6.3% (IV_seeds_PHY_PM)—28.48% (IV_seeds_PHY)] and *Enterococcus* [6.72% (IV_seeds_PHY)—12.87% (IV_seeds_PHY_PM)] was characteristic of all treatments, while their representation in the negative control was low (1.83% and 2.87%, respectively). Finally, treatment IV_seeds_PM stood out from the other samples due to the high relative abundance of Erwiniaceae (4.38%) and uncultured *Diplorickettsiaceae* (13.89%).

Figure S3 in the Supplementary Materials displays a corresponding heatmap of scaled average abundances of the 21 most prevalent bacterial phyla across all seed samples, including negative controls and treatments, before and after harvesting.

The most abundant taxa detected in poultry manure were *Staphylococcus* (27.55%), *Pseudogracilibacillus* (10.23%), and *Bacillaceae* (10.46%), followed by unc_*Bacillaceae* (5.87%), *Oceanobacillus* (4.02%), *Yaniella* (3.35%), *Jeotgalicoccus* (3.32%), and *Nocardiopsis* (2.61%). The rest of the genera with higher relative abundance (1.14–2.05%) in the tested poultry manure samples were *Salinicoccus*, *Gracilibacillus*, *Novibacillus*, *Virgibacillus*, *Thermoactinomyces*, *Melghirimyces*, *Atopostipes*, *Bacillus*, and *Corynebacterium*. The rest of the taxa were represented in less than 1% of abundance (Figure S4).

### 2.5. Differential Abundance Analysis

**Figure 6:**
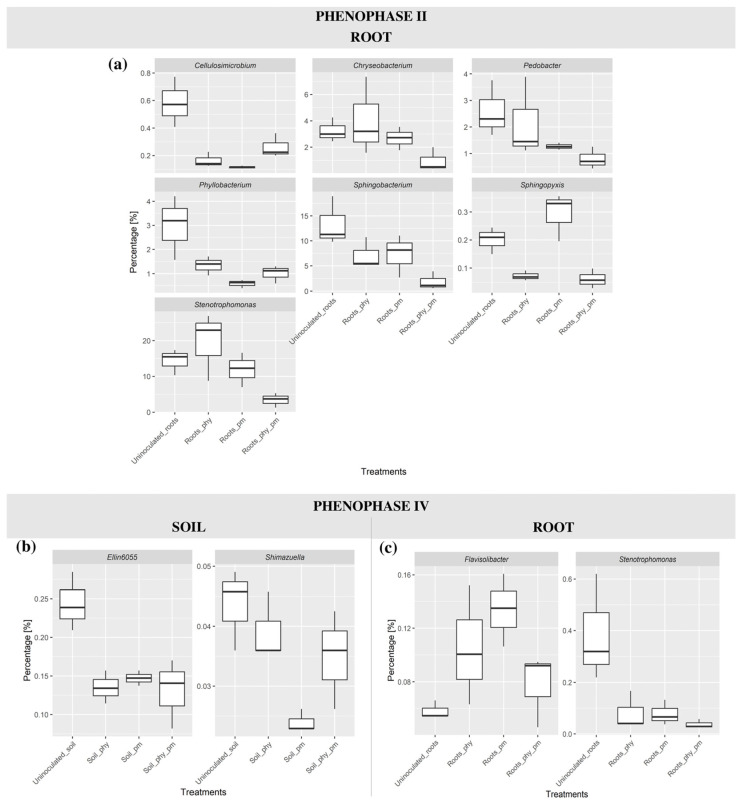
Differential abundance analysis results for (**a**) root samples collected during phenophase II and for (**b**) soil samples and (**c**) root samples collected during phenophase IV.

A differential abundance analysis identified statistically significant shifts in certain taxa across phenophases II–IV, comparing rhizosphere and root microbiota between treatment groups and the negative control (uninoculated soil). These results provide insights into how microbial communities respond to different treatments over time, highlighting taxa that are either enriched or depleted in response to inoculation. The results are presented in Figure 6 for phenophases II and IV, while a full table of the differential abundance analysis can be found in Table S2 of the Supplementary Materials.

Considering soil microbial dynamics, in phenophase II, no significant differences in taxa abundance were observed in the rhizosphere samples between any of the treatment groups and the negative control. However, by phenophase III, shifts in microbial composition became evident. Taxon *11-44* exhibited a statistically significant increase in abundance in rhizosphere samples treated with PM compared to the negative control, with a log_2_ fold change (logFC) of 1.4 (Table S2). By phenophase IV, further changes in microbial composition were detected. Notably, two taxa, *Ellin6055* and *Shimazuella*, showed a significant decrease in abundance in PM-treated soils compared to the negative control, with logFC values of −0.63 and −0.73, respectively. These negative values indicate that these taxa were more abundant in the negative control (Figure 6b).

Microbial shifts were also observed in root-associated microbiota across the different phenophases. In phenophase II, significant differences in taxa abundance were detected between PM-treated roots and the negative control. The taxa *Cellulosimicrobium* and *Phyllobacterium* were significantly less abundant in PM-treated roots, with logFC values of −1.71 and −1.70, respectively. A more pronounced shift was observed when comparing roots treated with PHY_PM to the negative control. Several taxa, including *Cellulosimicrobium*, *Chryseobacterium*, *Pedobacter*, *Phyllobacterium*, *Sphingobacterium*, *Sphingopyxis*, and *Stenotrophomonas*, exhibited significantly lower abundance in PHY_PM-treated roots, with logFC values of −1.70, −2.38, −2.12, −1.97, −3.14, −2.19 and −2.48, respectively. The strong negative logFC values indicate a substantial depletion of these taxa in the PHY_PM-treated samples (Table S2 and Figure 6a). In phenophase III, the root samples did not exhibit any statistically significant changes in microbial abundance between the treatment groups and the negative control. By phenophase IV, however, notable differences re-emerged. In the PM-treated roots, the taxon *Flavisolibacter* was significantly enriched compared to the negative control, with a logFC of 0.86. In contrast, in the PHY_PM-treated roots, the taxon *Stenotrophomonas* was significantly depleted, with a logFC of −2.11, indicating that it was more abundant in the negative control (Figure 6c).

### 2.6. Whole-Genome Sequencing of Bacillus subtilis sp. subtilis and Microbacterium sp.

Isolates of the main bacterial species of the phytobiotic formulation, *Bacillus subtilis* sp. *subtilis* and *Microbacterium* sp., previously characterized only based on 16S rRNA, were subjected to WGS, and assembled genomes were annotated, and phylogenetic trees were constructed to confirm their identity. After quality filtering, 3,156,291 and 2,192,459 paired-end reads were used for *B. subtilis* sp. *subtilis* and *Microbacterium* sp. genome assembly, respectively.

#### 2.6.1. *Bacillus subtilis* sp. *subtilis*

The de novo assembly of *B. subtilis* sp. *subtilis* resulted in 73 contigs, with an N50 length of 447,052 bp and a total genome size of 3,955,365 bp, with a GC content of 43.90%. Compared to phylogenetically related *B. subtilis* strains, the assembled genome is slightly shorter, while its GC content falls within the previously reported range (Table 1). A total of 4024 genes were predicted by Dfast, including 3956 coding DNA sequences, 1 rRNA, and 67 tRNAs.

**Figure 7:**
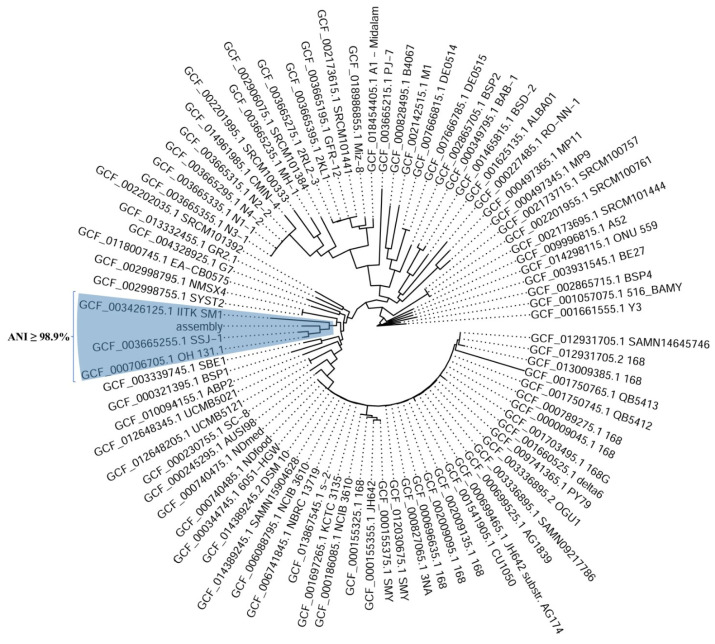
Phylogenetic tree of *Bacillus subtilis* sp. *Subtilis*, constructed using conserved marker genes.

The phylogenetic analysis used PhyloPhlAn to analyze multiple microbial genomes. The tool was configured to focus on highly variable sites, remove fragmented or low-quality entries, and construct a phylogenetic tree using conserved marker genes. The *Bacillus* BM-15a assembly was classified within the genus *Bacillus* and formed a homogenous cluster with strains SSJ1, OH 131.1, and IITK SM1 (Table 1 and Figure 7).

To determine the taxonomic position of *B. subtilis* sp. *subtilis BM-15a*, the assembled genome (assembly) was compared against multiple reference genomes of *Bacillus subtilis* sp. *subtilis* using average nucleotide identity (ANI). ANI values above 95% indicate that genomes belong to the same species, while ANI values >99.99% indicate the same strain. The analysis showed that the assembled genome shared the highest ANI value of 99.43% with the *Bacillus subtilis* strain OH 131.1 (GCF_000706705.1), followed closely by strain SSJ-1 (GCF_003665255.1) at 99.33% ANI. Additional comparisons revealed ANI values ranging from 98.90% to 98.81% with other *B. subtilis* strains, including IITK SM1, SBE1, and SC-8. All comparisons had over 1245 matched fragments out of 1305, indicating high genomic similarity across the tested strains (Table 2).

In contrast, the most distantly related genomes in the dataset are strains ALBA01, BAB-1, MP11, PJ-7, and RO-NN-1, with ANI values ranging from 97.86% to 98.15%. Although these values confirm that the isolate belongs to the *B. subtilis* species, they indicate a greater genomic distance compared to the top matches. Among these, strain RO-NN-1 had the lowest ANI value (97.86%), followed by PJ-7 (98.02%) and MP11 (98.08%) (Table 2).

A complete table of the comparative genomic analysis of *Bacillus subtilis* sp. *subtilis* assemblies, based on the average nucleotide identity, is available in the Supplementary Materials (Table S3).

When it comes to functional annotation and biocontrol potential, WGS of BM-15a was analyzed using NCBI’s Conserved Domain Database (CDD) to identify functional domains and key metabolic pathways. The genome annotation revealed the presence of loci encoding polyketide synthases (PKSs), including PksS, PksR, PksN, PksM, and PksL, as well as loci for serine protease (AprX) and bacillolysin. In addition to antimicrobial potential, the genome contains genes involved in siderophore biosynthesis, notably the bacillibactin exporter (YfmO) and its regulatory elements. The presence of glutamine synthetase (GlnA) and citrate synthase was also confirmed. Moreover, genes associated with oxidative stress response, including peroxiredoxin and oxidoreductases, were identified. Furthermore, an essential gene for IAA synthesis, anthranilate synthase, was found, along with genes encoding aminopeptidase AmpS, flagellin, and the exopolysaccharide biosynthesis protein EpsI.

#### 2.6.2. *Microbacterium* sp.

For *Microbacterium* sp., de novo assembly generated 104 contigs, with an N50 length of 44,752 bp and a total genome size of 2,953,076 bp, with a GC content of 70.08%. Relative to closely related *Microbacterium* species, the assembled genome is shorter, while the GC content remains consistent with previously reported values (Table 3). A total of 2748 genes were predicted by Dfast, including 2698 coding DNA sequences, 3 rRNAs, and 47 tRNAs.

**Figure 8:**
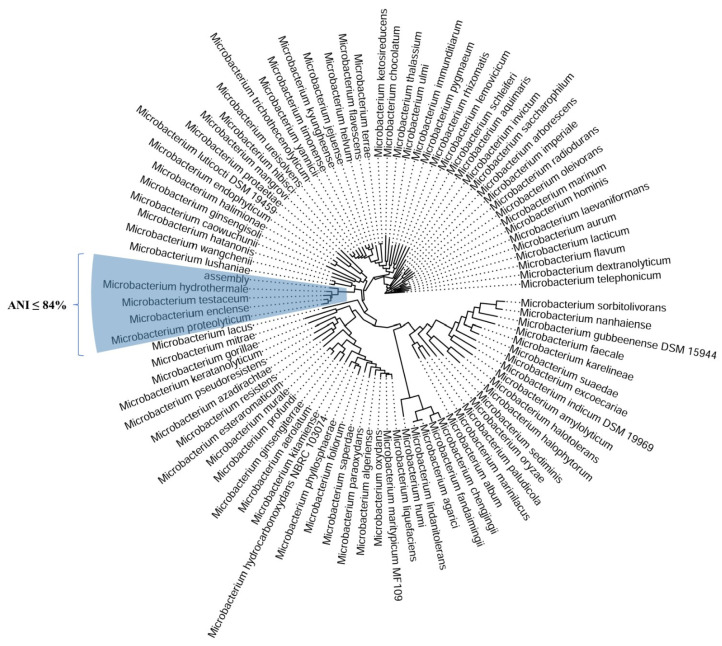
Phylogenetic tree of *Microbacterium* sp., constructed using conserved marker genes.

For both species, 99.5% genome completeness was obtained based on Busco results (0.2% out of 99.5% were associated with duplicated Busco genes). A phylogenetic analysis using PhyloPhlAn to analyze multiple microbial genomes was also performed for *Microbacterium* sp. The AL-11a assembly was classified within the genus *Microbacterium* and formed a homogenous cluster with the species *Microbacterium hydrothermale*, *Microbacterium testaceum*, *Microbacterium enclense*, and *Microbacterium proteolyticum* (Table 3 and Figure 8).

The ANI values observed for a comparison between the assembled genome and various *Microbacterium* species all fall well below the 95% threshold, indicating that the genome is positioned within the *Microbacterium* genus but is distinctly separated from the species of the tested strains. The highest ANI value, recorded with *Microbacterium hydrothermale* (GCF_004854025.1), was 84.04%, suggesting that this isolate represents a completely new species. Similarly, *Microbacterium enclense* (GCF_900096885.1) showed an ANI of 83.70%, further indicating a notable genetic divergence between this species and the assembled genome. ANI values for other *Microbacterium* species ranged from 80.44% to 84.04%, reflecting significant genetic separation, with the lowest ANI value being observed with *Microbacterium hibisci* (GCF_015278255.1) at 80.44% (Table 4).

A complete table of the comparative genomic analysis of *Microbacterium* sp. assemblies, based on the average nucleotide identity, is available in the Supplementary Materials (Table S4).

To further determine the taxonomic position of *AL-11a*, we calculated ANI by comparing its draft genome assembly with publicly available genomes of closely related *Microbacterium* species. These analyses provide a quantitative measure of genetic similarity, helping us to clarify its classification within the genus.

Functional annotation of the AL-11a genome revealed a broad range of genetic features that contribute to plant growth promotion, stress tolerance, and biocontrol activity. To enhance nutrient availability, the genome encodes genes for citrate synthase and glutamine synthetase (GlnA). Additionally, genes involved in iron acquisition, such as siderophore-interacting proteins, cysteine desulfurase for Fe-S clusters, iron-dicitrate transporters, MFS_YfmO_like proteins, and ABC transporter ATP-binding proteins, were identified. In terms of phytohormone production, AL-11a contains genes related to indole-3-acetic acid (IAA) biosynthesis, including anthranilate synthase and flavin-containing amine oxidase. The genome also includes genes for heat shock proteins (DnaK and DnaJ) and oxidoreductases, such as NADPH-dependent FMN reductase and alkanesulfonate monooxygenase. Notably, the bacterium carries genes for carotenoid biosynthesis, specifically phytoene synthase and phytoene dehydrogenase. Moreover, genes involved in exopolysaccharide synthesis, including phosphotransferases and glycosyltransferases, were identified. Potential biocontrol properties are supported by the presence of genes related to pathogen suppression, such as SGNH hydrolases and serine proteases. An additional significant feature is the presence of a tellurium resistance protein (TerC), along with genes for thiamine (vitamin B1) biosynthesis, including thiamine-phosphate synthase and hydroxyethylthiazole kinase.

### 2.7. Evaluation of Yield Parameters

**Figure 9:**
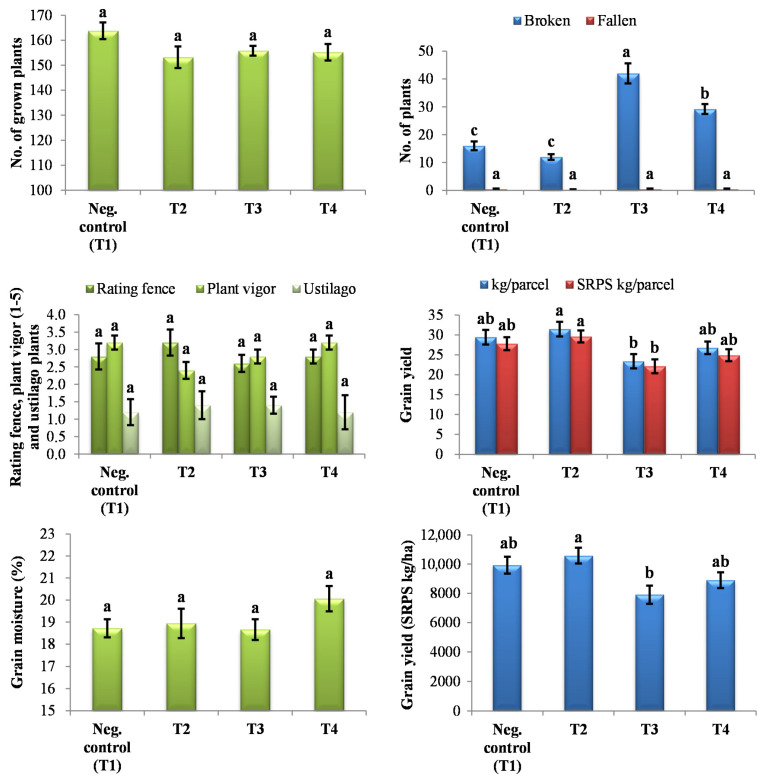
Histograms showing statistically processed values for the basic parameters (number of grown plants, rating fence, plant vigor, the occurrence of *Ustilago* sp., grain moisture, number of fallen/broken plants, and grain yield) obtained after harvesting in the experimental field. According to Tukey’s HSD test, all values marked with the same letter within the histogram are not statistically significant.

The results of the yield and associated parameters, evaluated after harvesting, are shown in Figure 9. Based on the obtained results, statistically significant differences in the values obtained for the tested parameters (number of grown plants, rating fence, plant vigor, the occurrence of *Ustilago* sp., and grain moisture) were not observed for any of the tested treatments (PHY, PM, and PHY_PM) in comparison to the negative control. However, the number of broken plants was statistically significantly higher in the treatments with PM and PHY_PM compared to the negative control and the treatment with PHY, showing a probability of *p* < 0.001 and *p* < 0.01, respectively. A statistically significant difference in the maize grain yield was observed in the treatment with PHY in comparison to the other two treatments (PM and PHY_PM) and the negative control. The lowest yield was obtained in the treatment with PM.

**Figure 10:**
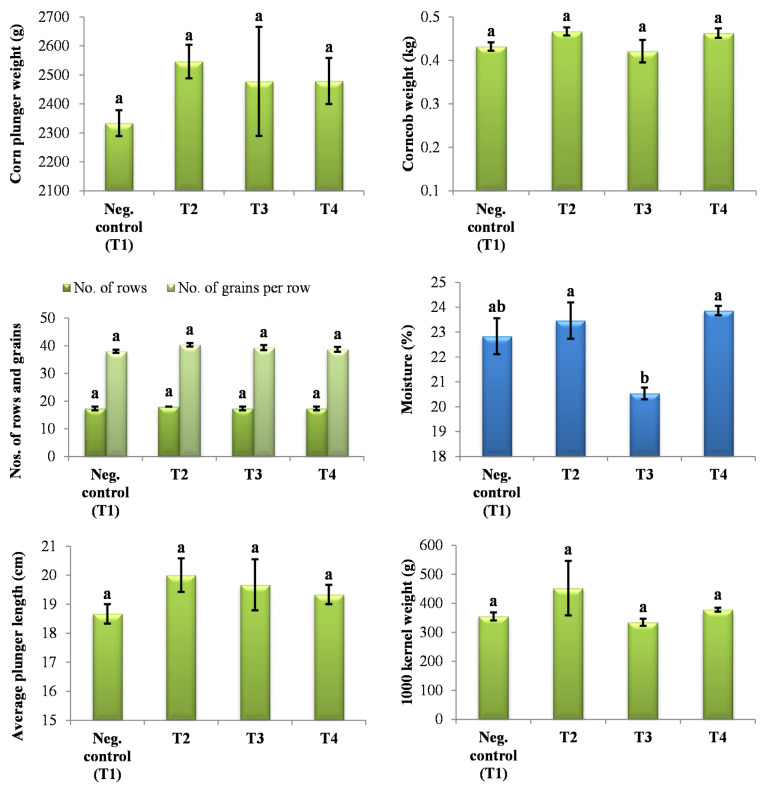
Histograms showing statistically processed values that were obtained for the maize cob parameters (maize plunger weight, number of rows and grains, average plunger length, corncob weight, moisture, and 1000 kernel weight) in the tested treatments. According to Tukey’s HSD test, values marked with the same letter within the histogram were not statistically significant.

The parameters measured for the maize plunger, depending on the treatment, are shown in Figure 10. Statistically significant differences in the maize plunger weight, number of rows and grains, average plunger length, corncob weight, and 1000 kernel weight were not observed regardless of the applied treatment. Based on the obtained results, seed moisture values were the highest and statistically significant in treatments with PHY and PHY_PM, while the lowest values for the grain moisture were obtained for the treatment with PM.

**Figure 11:**
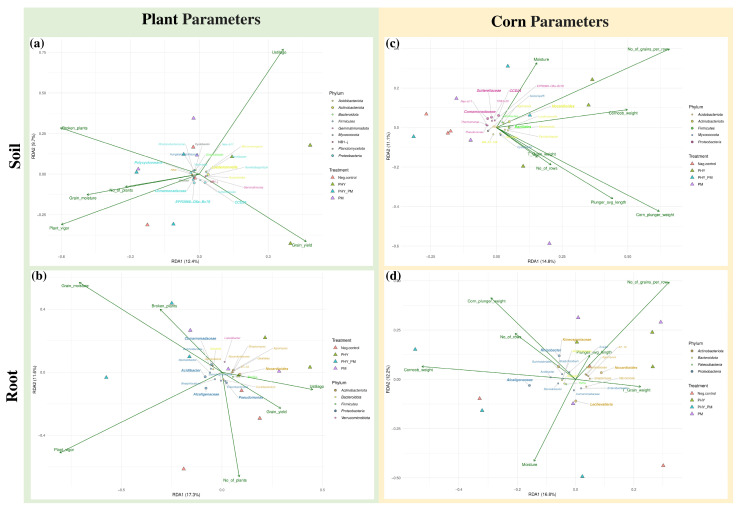
Redundancy analysis (RDA) of relationships between the 20 most influential genera in bacterial community structure and plant traits in maize rhizosphere and root samples. (**a**) RDA biplot of soil bacteriobiota and general plant performance traits. (**b**) RDA biplot of root bacteriobiota and general plant performance traits. (**c**) RDA biplot of soil bacteriobiota and maize cob parameters. (**d**) RDA biplot of root bacteriobiota and maize cob parameters.

RDA was used to assess the relationship between the maize plant performance parameters and bacterial community composition in both the soil and root samples at the harvesting stage (Figure 11). The analysis focused on bacterial communities shaped by four treatment conditions. Overall, the RDA revealed that plant vigor was positively correlated with the negative control (T1) and T4 (PHY_PM), grain yield with T1 and T2 (PHY), grain moisture with T3 (PM) and T4, and the number of plants with T3 and T4. In contrast, the number of broken plants was negatively correlated with T1 and T2, while *Ustilago* showed a negative association with T4.

When evaluating correlations between plant traits and soil bacteriobiota, grain yield showed a positive correlation with *CCD24*, *Sutterellaceae*, and *Noviherbaspirillum* and a negative correlation with *Cystobacter*, *Variovorax*, and *Dinghuibacter*. Plant vigor correlated positively with *Comamonadaceae* and *Polycyclovorans* and negatively with *Noviherbaspirillum* and *Micromonospora*. The number of broken plants was inversely related to grain yield and positively correlated with *Hungateiclostridiaceae*. Grain moisture was positively correlated with *Cystobacter*, while the number of plants showed positive correlations with *EPR3968-O8a-Bc78*, *RB41*, and *Neo-b11* and negative correlations with *Luedemannella*, *Nocardioides*, and *Gemmatimonas*. *Ustilago* infection was negatively correlated with *EPR3968-O8a-Bc78* (Figure 11a). Spearman’s rank correlation analysis confirmed statistically significant correlations (FDR < 0.1) between *EPR3968-O8a-Bc78* and *Ustilago*, *Hungateiclostridiaceae* and the number of broken plants, and *Dinghuibacter* and grain yield (Table S5).

For root-associated bacteriobiota, plant vigor was positively associated with *Alcaligenaceae*, *Acidibacter*, *Lechevalieria*, *Steroidobacter*, and *Bradyrhizobium* and negatively associated with *Luteolibacter*, *Nocardioidaceae*, and *Agromyces*. Grain yield was positively correlated with *67-14*, *Streptomyces*, and *Bacillus*. The number of grown plants was positively correlated with *Pseudomonas*, *Enterobacterales*, and *Gaiellales*. The number of broken plants was positively correlated with *Niastella* and negatively with *Streptomyces* and *Bacillus*. Grain moisture was positively correlated with *Comamonadaceae*, *Burkholderiales*, and *Niastella* and negatively with *Stenotrophomonas* and *Curtobacterium* (Figure 11b). However, only a few correlations reached statistical significance: plant vigor showed significant positive correlations with *Acidibacter* and *Bradyrhizobium* and grain moisture with *Comamonadaceae* (Table S5).

Regarding the maize cob parameters, while several apparent associations with bacterial taxa were observed, none of the adjusted *p*-values from correlation tests were below 0.1 for either soil or root datasets, indicating no statistically significant correlations between the rhizosphere/root microbial communities and these specific cob traits (Figure 11c,d).

## 3. Discussion

In the pursuit of sustainable agricultural practices, the use of organic solutions such as poultry manure and seed coating with beneficial microorganisms has gained increasing attention [37,38]. These strategies aim to enhance soil fertility and plant health while reducing reliance on synthetic fertilizers and pesticides. Seed coating with plant growth-promoting bacteria offers a targeted approach to stimulate early plant development and shape the rhizosphere microbiome, while poultry manure serves as a rich organic input that supports microbial proliferation and nutrient cycling [11,39]. The combined application of these approaches presents a promising avenue for improving crop productivity and maintaining soil microbial stability in a more environmentally conscious way [40]. Building on these sustainable approaches, our study investigated how poultry manure and seed-applied beneficial bacteria influence the maize microbiome and its dynamics throughout plant development.

When it comes to bacterial alpha diversity, in both soil and root samples, there was a noticeable progressive increase throughout the plant’s developmental stages. These results suggest that as maize plants mature, their roots and the surrounding rhizosphere become increasingly colonized by a broader range of microbial taxa. This trend is likely caused by changes in root exudation profiles, nutrient availability, and soil microenvironment conditions as the plant develops [41,42]. A recent study by Bourceret et al. [43] also detected an increase in the bacterial alpha diversity in the soil and rhizosphere of maize from the vegetative to the reproductive growth phases but in the root compartment diversity, remained stable over growth stages.

The increased rhizosphere microbial richness observed in PM treatments can be attributed to the immediate influx of nutrients, as poultry litter application significantly alters soil chemistry and enriches certain bacterial groups. However, this benefit diminishes in later stages [44,45]. A similar trend was observed in taxa such as *Sphingomonas*, *Microvirga*, *Streptomyces*, and *Nocardioides*, which initially increased in abundance in soil treated with PM compared to a negative control but declined in subsequent phenophases. Notably, these taxa were not abundant in the poultry manure itself. The initial nutrient surge provided by the manure appears to have stimulated indigenous soil populations—such as *Sphingomonas* and *Streptomyces*—that can metabolize manure-derived substrates. Their temporary increase likely reflects the activation of these native soil communities rather than a direct introduction from the manure [46,47,48,49].

In contrast, initially, T2 root samples exhibited reduced microbial diversity, suggesting that the introduced strains may have outcompeted or occupied niches of some native microbial populations. However, in later phenophases, T2 displayed the highest evenness, indicating a more balanced microbial community, possibly due to the inoculant’s influence over time [50].

The combined application of PM and beneficial inoculants consistently enhanced microbial diversity and evenness in the rhizosphere across all growth stages, suggesting that this synergy fosters favorable conditions for microbial proliferation and ecological balance. In root samples, a similar trend was observed for T4, except during phenophase IV, where a slight decline in diversity metrics was noted compared to other groups. This may indicate a shift toward a more specialized microbial community due to prolonged selection pressures from both the inoculants and organic fertilizer [51].

When considering seeds harvested in phenophase IV, the higher richness and Shannon index in T4 can be connected to the combined effect of PM and inoculants. The introduction of *Bacillus subtilis* sp. *subtilis* BM-15a and *Microbacterium* sp. AL-11a into PM likely enhanced microbial interactions in the soil, promoting a more diverse and complex microbial community that could colonize the seeds [52,53]. Additionally, PM provided an organic nutrient source, further supporting microbial diversity by fostering a rich soil microbiome that could be transferred to the developing seeds [45]. On the other hand, the highest Pielou’s evenness in T3 suggests a more balanced microbial composition within these seeds. Unlike T4, where inoculated bacteria could have led to the domination of certain microbial species, T3 likely allowed for a more balanced distribution of microbial species. This suggests that while PM encouraged microbial diversity, it did so in a way that maintained a more even distribution across different taxa [54].

The applied treatments (PHY, PM, and their combination) during the four tested phenophases did not affect the composition of the indigenous soil bacterial communities. It is highly important that for the selection of suitable measures to maintain soil stability, the application of different agricultural practices must not have a negative impact on soil microbiome composition. An earlier study also reported that the application of bioorganic fertilizers has shown to maintain a more stable soil microbiome than applied chemical fertilizers [55]. On the other hand, root bacterial communities differ in distribution and relative abundance between phenophases and treatments. The gradual shift in microbiota composition throughout maize growth could be related, in the first place, to the production of different root metabolites over the growing season [43].

In the seedling stage, roots were rich in plant-associated genera *Stenotrophomonas*, *Sphingobacterium*, and *Achromobacter*, including *Pseudomonas* and *Serratia*, known as phosphate solubilizers [56]. In the treatments (PHY, PM, and PHY + PM), an increase in *Enterobacteriaceae* abundance was evident. This could be a beneficial shift for the treated maize, since the *Enterobacteriaceae* family includes the plant growth-promoting representatives *Klebsiella* spp. and *Enterobacter* spp. as nitrogen fixators and phosphate solubilizers [57]. The application of phytobiotic formulations and poultry manure together potentially modified the production of the root exudates in a way to attract and promote a higher presence of *Streptomyces*, *Lechevalieria*, *Agromyces*, *Pantoea*, *Nocardioides*, and *Sphingomonas* compared to the other treatments. Furthermore, in the flowering phenophase, *Bacillus* prevailed in the analyzed communities. There is a wide range of beneficial effects of *Bacillus* for plants, including phosphate solubilizing, growth hormone biosynthesis, antifungal metabolites, inducing systemic resistance, etc. [58]. In the harvesting phenophase (IV), the relative abundance of the most significant genera in roots did not vary much between the treatments, reaching the stable microbiome related to maize [59]. The most abundant genera in the harvesting phenophase was *Lechevalieria*, which is positively correlated with root length and vitality, promoting healthy growth [60]. Seeds were characterized by lower bacterial diversity compared to the soil, root, and manure. In pre-harvest seeds treated with PHY, the most abundant genus was *Acinetobacter*, followed by *Pantoea*, *Pseudomonas*, and *Bacillus*. The impact of the applied PHY was highly beneficial considering the PGP abilities of these genera and also that they were confirmed to be core members of maize in many studies [56]. As core members, the genera *Pantoea* and *Pseudomonas* were still detected after harvesting. As was detected earlier for root bacterial communities in seedling phenophases, representatives of *Enterobacteriaceae* were the most abundant in the combined treatment (PHY + PM) of seeds, indicating that their potential colonization of seeds was stimulated with applied treatment. Besides the presence of *Bacillaceae*, poultry manure was characterized by a high abundance of *Staphylococcus* and *Pseudogracilibacillus*, genera that were not further detected in the maize bacteriobiome. Generally, *Pseudogracilibacillus* sp. was isolated earlier from the rhizosphere of maize [61] and plant roots [62].

A differential abundance analysis revealed dynamic shifts in the maize rhizosphere and root microbiomes in response to treatments across phenophases. While no significant changes were observed in rhizosphere microbiota during phenophase II, differences became apparent in phenophase III and IV, with certain taxa being selectively enriched or depleted depending on the treatment applied. By the harvesting phase, the oligotrophic taxa *Ellin6055* and *Shimazuella* declined significantly in the PM-treated soils. These slow-growing soil bacteria, adapted to low-nutrient conditions, are known for their roles in pollutant degradation, nutrient cycling, and plant protection [63,64,65]. The observed decline in these taxa during the final phenophase may reflect a delayed ecological response to early-season PM application. As noted by Fierer et al. [66], nutrient pulses from organic amendments can initially promote copiotrophic taxa at the expense of oligotrophs, even after nutrient levels subside.

Root-associated communities exhibited greater sensitivity to treatment. In phenophase II, *Cellulosimicrobium* and *Phyllobacterium*, both beneficial endophytes with plant growth-promoting and biocontrol functions, were significantly reduced in PM-treated plants. Such declines are consistent with reports that long-term manure use can suppress specific microbial groups [67,68,69]. The reduced abundance of several key genera in the PHY_PM-treated roots—including *Cellulosimicrobium*, *Chryseobacterium*, *Pedobacter*, *Phyllobacterium*, *Sphingobacterium*, *Sphingopyxis*, and *Stenotrophomonas*—suggests that this treatment may disrupt root microbial networks. These genera support plant health through nutrient mobilization, phytohormone production, antimicrobial activity, and the induction of systemic resistance [37,40,67,68]. Their decline could result from competitive exclusion or changes in root exudation triggered by the treatment [70,71]. This bacterial shift may have both beneficial and disadvantageous consequences. On the one hand, the reduced abundance of genera such as *Chryseobacterium*, *Pedobacter*, and *Stenotrophomonas* may lower the presence of opportunistic pathogens or taxa known to harbor antibiotic resistance genes in the soil environment [72,73,74]. On the other hand, the suppression of beneficial taxa—such as *Phyllobacterium* and *Sphingopyxis*, which are associated with nitrogen fixation, and *Sphingobacterium*, which contributes to the degradation of complex xenobiotics—could compromise nutrient cycling and stress resilience. Additionally, while *Stenotrophomonas* includes potentially pathogenic strains, it also functions as a PGPR in some systems. Therefore, its decline may represent a trade-off between pathogen suppression and the loss of potential plant-beneficial functions [75,76,77]. No significant changes were observed in root microbiota during phenophase III, suggesting a temporary microbial equilibrium. However, at the harvesting stage, a significant enrichment of the genus *Flavisolibacter* was detected in PM-treated roots compared to the negative control. *Flavisolibacter* is known for its resistance to drought stress and its ability to improve both the physical and biological properties of rhizosphere soil [78]. In contrast, a significant depletion of *Stenotrophomonas* was observed in the PHY_PM-treated roots relative to the control. As a plant growth-promoting rhizobacterium, *Stenotrophomonas* contributes to nitrogen fixation, ammonia, and indole-3-acetic acid production and supports key biochemical processes that enhance plant growth and maintain soil health [79]. The observed reduction in beneficial taxa during the seedling and harvesting phases suggests potential trade-offs associated with organic and bio-based treatments on the composition of root-associated microbial communities.

An analysis of beta diversity also revealed that the applied treatments had an influence on the structure of bacterial communities, as was also confirmed by a study by Kusstatscher et al. [80]. Notably, the samples from phenophase IV clustered together regardless of treatment, suggesting a more stable microbial profile at this stage. Meanwhile, phenophases II and III displayed more diverse microbial compositions, with distinct clustering patterns influenced by treatment and maize developmental stage.

In light of public requests for healthier food protection and production, formulating biofertilizers for the enhancement of crop yields is of high interest. The present study evaluated microbial inoculants as a consortium of two beneficial strains, BM-15a and AL-11a. The treatment with PHY induced statistically higher values of maize grain yield and seed moisture, whilst the number of broken plants was at the level of the negative control. As an active component of biofertilizers, *Bacillus subtilis* was previously confirmed to be a promoter of the growth and development of maize seedlings when applied in the seed-coating technique [39]. The benefits of *Bacillus*-based fertilizers’ application to soil promotes crop yields through the enhancement of plant-available forms of nutrients, the inducement of pest defense systems, and the control of different pathogenic microbes [81]. Besides previous findings of *Microbacterium* sp. as PGP bacteria [82], the higher maize yield in our case could not be related to this beneficial microorganism, since appropriate ASVs were not detected during the analyzed phenophases. The absence of *Microbacterium* sp. could be related to the seed coating step, which probably failed to be firmly attached to the maize grain.

The RDA and correlation analyses revealed several patterns in the relationships between bacterial taxa and plant performance traits; however, only a limited number of these associations remained statistically significant after correction for multiple tests. Among the soil-associated taxa, *EPR3968-O8a-Bc78*, *Hungateiclostridiaceae*, and *Dinghuibacter* exhibited significant correlations with *Ustilago* infection, the number of broken plants, and grain yield, respectively.

*Dinghuibacter* has been identified in rhizosphere microbial communities of invasive plant species, such as *Ambrosia artemisiifolia* (common ragweed), where it has been implicated in enhanced carbon and nitrogen cycling and improved phosphorus mobilization in the rhizosphere. These functions likely support nutrient acquisition and rapid plant growth in competitive environments [83]. In this study, the significant negative correlation between *Dinghuibacter* abundance and maize grain yield suggests a potential competitive interaction, whereby the metabolic activity of *Dinghuibacter* may hinder nutrient availability or otherwise negatively impact plant productivity. A significant positive correlation was observed between the abundance of *Hungateiclostridiaceae* and the number of broken maize plants, particularly under poultry manure (PM) treatments. Although the ecological role of this bacterial family is not well-characterized, the available literature reports its enrichment in farmyard manure-treated soils, where members of this family have been described as producers of cellulolytic and xylanolytic enzymes [84]. *Hungateiclostridiaceae* belongs to the order *Clostridiales*, a group of anaerobic bacteria involved in the decomposition of complex organic matter, leading to the generation of gases and organic acids [85]. These intensified microbial decomposition processes may alter the physical structure of the soil—such as decreasing aggregate stability or increasing porosity—which could compromise root anchorage and stem support, making plants more prone to mechanical damage or breakage [86].

In the root compartment, statistically significant positive correlations were found between plant vigor and the genera *Acidibacter* and *Bradyrhizobium* and between grain moisture and *Comamonadaceae*. Both *Acidibacter* (a member of the phylum *Acidobacteria*) and *Bradyrhizobium* are known for nodule formation and their plant growth-promoting properties, including the stimulation of root elongation and lateral root development, which can enhance nutrient and water uptake and thereby contribute to improved plant vigor [87,88]. *Comamonadaceae*, commonly found in rhizosphere environments, are known participants in sulfonate cycling and the degradation of organic compounds, and they play an important role in nutrient cycling, which can influence soil moisture retention and availability [89]. Notably, all three taxa were particularly abundant in the PHY_PM-treated samples and in the negative control group.

Overall, the relatively low number of statistically significant correlations—especially in relation to cob traits—suggests that the influence of bacterial communities on plant performance is complex and likely modulated by additional biotic and abiotic factors not fully accounted for in the present study.

To verify the identity of the two beneficial bacterial strains used for seed coating, a whole-genome ANI analysis was performed, offering greater taxonomic resolution than 16S rRNA sequencing. The *Bacillus* isolate, initially identified as *Bacillus subtilis* sp. *subtilis* BM-15a, exhibited the greatest similarity to the strain OH 131.1 (ANI = 99.43%). Although this value confirms species-level identity, it falls below the 99.99% threshold typically used for strain-level classification, indicating that the isolate may represent a distinct, closely related strain. The lowest observed ANI value (97.86%) with strain RO-NN-1 further supports strain-level divergence, which could reflect ecological adaptation or genomic differences relevant to its functional role in the rhizosphere [90].

Whole-genome annotation of the *B. subtilis* isolate revealed multiple plant-beneficial traits. Biosynthetic gene clusters for polyketide synthases suggest the production of antimicrobial compounds that are effective against pathogens such as *Fusarium* and *Rhizoctonia* [91,92,93]. Enzymes like serine protease (AprX) and bacillolysin further contribute to fungal cell wall degradation, reinforcing biocontrol potential [94,95]. The detection of siderophore biosynthesis genes, including the bacillibactin exporter YfmO, indicates an enhanced iron acquisition capacity, conferring competitive advantages in the rhizosphere and limiting iron availability to pathogens [96,97]. The presence of glutamine synthetase (GlnA), molybdopterin synthase (MoaD/E), and citrate synthase supports nutrient mobilization through nitrogen assimilation and phosphate solubilization, particularly under alkaline or nutrient-poor conditions [98,99,100]. The bacterium also possesses oxidative stress response mechanisms, including peroxiredoxin and oxidoreductases, enhancing survival during plant interactions [101,102,103]. Additionally, anthranilate synthase and flavin-containing amine oxidase indicate the potential for indole-3-acetic acid (IAA) synthesis via the tryptamine pathway, contributing to root development and plant stress tolerance [104]. The presence of aminopeptidase AmpS and flagellin further implies roles in soil nutrient cycling and the induction of systemic resistance in plants [105,106]. Furthermore, the presence of exopolysaccharide biosynthesis genes, such as epsI, highlights the bacterium’s ability to form protective biofilms, improving plant tolerance to abiotic stress [107]. Together, these genomic features position *B. subtilis* sp. *subtilis* as a multifunctional plant growth-promoting bacterium with strong potential for use in biocontrol and sustainable agriculture.

In parallel, an ANI analysis of the *Microbacterium* isolate revealed all values below the 95% species-level threshold, with the closest match—*Microbacterium hydrothermale*—sharing only 84.04% identity. This confirms the isolate as a novel species within the *Microbacterium* genus. Functional genome annotation indicated similar plant-beneficial traits to those observed in the *Bacillus* strain, though executed through distinct genetic strategies. Nutrient acquisition capabilities in AL-11a are mediated by genes for phosphate solubilization (citrate synthase), iron uptake (siderophore-interacting proteins, Fe-S cluster assembly proteins, and ABC transporters), and nitrogen metabolism (glutamine synthetase), collectively supporting improved plant nutrient availability and suppression of phytopathogens through competitive exclusion [96,98,100]. Like *B. subtilis*, this strain also appears to produce IAA via the tryptamine pathway, as evidenced by the presence of anthranilate synthase and flavin-containing amine oxidase, reinforcing its role in root growth promotion and stress modulation [104,108]. Abiotic stress tolerance is conferred by a suite of genes encoding heat shock proteins (DnaK and DnaJ), oxidoreductases, and sulfur-metabolizing enzymes such as alkanesulfonate monooxygenase, enabling adaptation to drought, oxidative stress, and temperature fluctuations [109,110,111,112,113,114]. Carotenoid biosynthesis genes (phytoene synthase and dehydrogenase) provide additional protection against environmental stressors, particularly oxidative damage from UV exposure [115]. Genes for exopolysaccharide and glycosyltransferase enzymes promote biofilm formation, enhancing rhizosphere persistence and plant protection under abiotic stress [107]. Furthermore, *AL-11a* harbors genes encoding SGNH hydrolases and serine proteases, which can disrupt pathogen biofilms and degrade fungal cell walls, thus strengthening its biocontrol potential [94,116,117]. The detection of the terC gene suggests a capacity for resistance to toxic metals such as tellurium, implying a role in bioremediation of contaminated soils [118]. Additionally, thiamine (vitamin B1) biosynthesis genes, including thiamine-phosphate synthase and hydroxyethylthiazole kinase, may contribute to plant metabolic health, particularly in nutrient-deficient environments [119].

Together, these two bacterial strains—*Bacillus subtilis* subsp. *subtilis* and a novel *Microbacterium* species—demonstrate complementary plant growth-promoting traits. Genes involved in phytohormone biosynthesis support improved nutrient and water uptake, contributing to enhanced biomass accumulation and grain yields, particularly under suboptimal conditions. The presence of oxidative stress response genes enhances the bacteria’s survival in challenging environments and may also improve plant tolerance to drought, salinity, and oxidative stress. Additionally, genes responsible for exopolysaccharide biosynthesis facilitate biofilm formation, promoting stable and persistent root colonization under field conditions. Collectively, these features reflect a synergistic functional potential, enabling the strains to enhance nutrient mobilization, boost stress resilience, and contribute to biocontrol, making them well-suited for application in sustainable agriculture. By promoting efficient root colonization, stabilizing the rhizosphere microbiome, and supporting plant development under both optimal and stress conditions, these strains offer a promising strategy for reducing chemical inputs in maize cultivation. Their integration as a seed coating represents a practical, scalable approach to sustainable agriculture, contributing to yield stability, resource efficiency, and improved resilience of cropping systems.

These findings emphasize the potential of combining organic fertilizers and microbial seed coatings as complementary strategies to boost crop performance while supporting soil microbial health. The observed effects on microbial diversity, community structure, and functional potential indicate that these treatments can reshape the plant microbiome in a beneficial manner. Future research should aim to optimize application methods and investigate long-term field performance across different environmental conditions and crop types. Additionally, improving the formulation and delivery of microbial inoculants—particularly to ensure consistent colonization—remains a critical step toward their broader agricultural implementation. This study adds to the growing evidence that sustainable farming methods can improve both crop yields and environmental health, helping to create more resilient and eco-friendly agricultural systems.

## 4. Materials and Methods

### 4.1. Agricultural Field Conditions and Treatments

An experiment with different seeds/soil treatments was performed in the experimental field (252 m^2^ in size, 44.941496582991874, 20.723514764605284) of the Research and Development Institute “Tamiš” located in Pančevo (Serbia) during April–September. The soil type at the experimental field was carbonate chernozem, with soybean being the primary crop. Primary fertilization was carried out with nitrogen, phosphorus, and potassium (NPK) (15:15:15; 250 kg/ha) and stabilized ammonium nitrate (SAN; 150 kg/ha). The agrochemical characteristics of the carbonate chernozem were as follows: hummus, 4.3%; pH in H_2_O_2_, 7.2; pH in KCl, 7.61%; available P_2_O_5_ content, 28.5 mg/100 g; available K_2_O content, 27.2 mg/100 g; and CaCO_3_, 13.1%. The concentrations of macro- and micronutrients (in µg/g) were as follows: S—1103.5, P—1488.4, K—31,124.1, Mg—14,102.9, Na—932.4, Fe—21,211.4, Ca—45,421.6, Al—18,535.2, Zn—30.1, Sr—36.1, Pb—7.3, Ni—21.1, Mn—605.5, Li—45.2, Cu—16.4, Cr—16.2, Co—7.6, Cd—0.92, Ba—41.5, and As—1.1. During the experiment, the soil was fertilized with 300 kg/ha of NPK at a ratio of 16:16:16. The hybrid maize cultivar ZP666 was used for a field trial treated with fungicide from the Maize Research Institute Zemun Polje. In total, three treatments and negative controls were included: (a) uninoculated seeds without poultry manure (PM) (T1—negative control); (b) coated seeds with a phytobiotic formulation (PHY; the formulation of two strains, *Bacillus subtilis* sp. *subtilis* BM-15a and *Microbacterium* sp. AL-11a, each at a concentration of 1 × 10^7^) and without PM (T2); (c) uninoculated seeds with PM (commercially available finished product)-supplemented soil (T3); and (d) uninoculated seeds in soil with PM, previously inoculated with PHY (T4). Commercially available poultry manure was used in treatments T3 and T4. According to the manufacturer’s specifications, it contained 4% total nitrogen, 3% organic nitrogen, 4% available P_2_O_5_, 4% available K_2_O, 27% organic carbon, and 8% humic acids and had a pH of 7.34.

### 4.2. Sample Collection and Measurements

The entire experiment was carried out at four different growth stages of maize. The experiment was performed on a 1 ha field with homogenous soil properties, which was divided into four plots (each with 2500 m^2^), and each plot was further divided into 5 equally sized plots. The middle of three replications was used for plant material and soil sampling. To minimize border effects, only the center of the plot of each experimental plot was used for sample collection (in total, 18 rows were sown on each plot, and the 9 central rows were sampled).

For DNA extraction and metabarcoding, the samples were taken in the following order. Before sowing (phenophase I), samples included uninoculated seeds, uninoculated soil, and PM inoculated with PHY. During the growing season, at the seedling V3 stage (phenophase II), flowering VT stage (phenophase III—approximately two months after sowing), and harvesting R6 stage (phenophase IV), both root and rhizosphere soil samples (defined as that tightly attached to the roots) were collected for each of the treatments and negative controls. Additionally, at phenophase IV, maize seeds were sampled from cobs of all treatments after harvesting. For rhizosphere and root sampling, three individual maize plants and the corresponding rhizosphere soil from the middle of the plots were randomly selected from each plot; these triplicate samples per plot were then pooled and thoroughly homogenized to yield one biological sample. This longitudinal design produced nine samples per treatment. Seeds were collected after harvesting (approximately 10 g of kernels per cob and pooled—three cobs formed one replicate; *n* = 3) and before sowing from different commercially purchased bags (approximately 10 g per replicate; *n* = 3) for DNA extraction and metabarcoding analysis. All samples were transported to a laboratory under cool conditions and stored at 80 °C prior to DNA extraction.

Furthermore, statistical analysis of processed values for the basic crop parameters (number of grown plants, rating fence, plant vigor, the occurrence of *Ustilago* sp., grain moisture, number of fallen/broken plants, and grain yield) and maize cob parameters (maize plunger weight, number of rows and grains, average plunger length, maize cob weight, number of grain rows, moisture, and 1000 kernel weight) in the tested treatments compared to the control samples, obtained after harvesting in the experimental field, was performed. The obtained values for all parameters were used for testing variance significance among the samples by Tukey’s HSD post hoc test (one-way ANOVA). The statistical significance applied in all tests was *p* < 0.05. Statistical analysis was performed using the software program IBM SPSS Statistics v. 23 (IBM SPSS Inc., Armonk, NY, USA).

### 4.3. DNA Extraction and Amplicon Sequencing Preparation

Samples were taken at different stages to assess bacterial diversity throughout the maize growth cycle (see Section 2.2). The maize seeds were rinsed from the fungicide and sterilized by washing with sterilized water, 70% ethanol (*v*/*v*) for 5 min, 3.5% sodium hypochlorite (*w*/*v*) for 10 min, and final washing in deionized water. The particles of the rhizosphere soil samples were separated from the roots to produce the root rhizosphere, which was vigorously mixed with a vortex. The remaining root samples were mechanically cut out and transferred to fresh tubes after surface sterilization as for seed sterilization.

The seeds, soil, and roots were homogenized with liquid nitrogen, and the ZymoBIOMICS^TM^ DNA Mini Kit D4300 (Zymo Research, Irvine, CA, USA) was used for DNA extraction, according to the manufacturer instructions. Amplification of V4 16S rRNA was performed with 515F [120] and 806R [121]. The primers 806R and 515F were elongated with the P1 adapter and with the sequence of linker, barcode, and adapter at their 5′ end, respectively, to generate amplicon libraries for sequencing on the Ion S5 System (Thermo Fisher Scientific, Woodlands, Singapore). The order of the samples on a PCR plate was randomized to reduce potential batch effects of PCR reactions. Each sample was amplified with a unique barcode in triplicates to reduce PCR bias. The PCR reaction mix and temperature profile were set as described in the Earth Microbiome Project [122] with the following modifications: Peptide nucleic acid (PNA) PCR clamps, PNAm and PNAp, were added to suppress plant plastid and the mitochondrial 16S rRNA gene at a concentration of 1.25 µM. Prior to use, the PNAs were incubated 10 min in a water bath at 80 °C, as suggested by the manufacturer (Eurogentec, Seraing, Belgium). A PNA annealing step was added to the PCR temperature profile, i.e., 78 °C for 10 s after the denaturation step [123]. PCR products, including negative controls, were checked on an agarose gel, and an equal volume of pooled triplicates was joined in a final library. The final library was cleaned with Agencourt AMPure XP magnetic beads (Beckman Coulter, Brea, CA, USA) using a bead-to-DNA ratio of 0.7:1. The quality of the DNA library and DNA concentration was determined by an Agilent 2100 Bioanalyzer using the High Sensitivity DNA Assay kit (Agilent Technologies, Waldbronn, Germany). The Ion 530^TM^ chip with the DNA library and the Ion S5^TM^ calibration standard were prepared with the Ion OneTouch^TM^ 2 system using the kit Ion 520^TM^ and Ion 530^TM^ Kit—OT2, and sequencing was performed on the Ion S5^TM^ System (Thermo Fisher Scientific, Carlsbad, CA, USA).

### 4.4. Raw Data and Statistical Analyses

Reads were analyzed with QIIME2 v. 2023.5 [124]. Sequences with forward and reverse primers were extracted with the “qiime cutadapt trim-single” method, and primers were trimmed [125]. Only full-length 16S rRNA V4 amplicons were retained in the dataset. DADA2 [126] implemented in the “qiime dada2 denois-pyro” method was used for denoising and for the determination of ASVs (amplicon sequence variants) with no additional trimming. A taxonomy classification was made with the feature classifier plugin [127]. The naive Bayes classifier [128] was trained with amplicon region specific sequences extracted from the SILVA reference database, version 138.1, with representative sequences at 99% identity [129]. The reference database was prepared with RESCRIPt [130]. ASVs assigned to chloroplast and mitochondrial DNA as well as ASVs not assigned to the Bacteria kingdom were removed. Qiime2 artefacts were used to create a phyloseq object with the qza_to_phyloseq (qiime2R, v. 0.99.6) function. Rarefaction was performed to an even depth of 34,840, 30,588, 12,000, and 55,402 reads for roots, soil, seeds, and manure, respectively. NAs were replaced with the last determined taxonomic level using the tax_fix (microViz, v. 0.12.6) function. Similarly, ASVs determined as “uncultured” were replaced with the last known taxonomic level (abbreviation “unc” was added next to the last known taxa group). Collapsing to the genus level was performed with the tax_glom_wt (ggClusterNet, v. 0.1.0) function.

Alpha diversity indexes (Shannon, richness, and Pielou evenness) were calculated with a vegan R package, v. 2.6-10. Differences between soil, roots, and seeds from different treatments and different sampling points were tested with the Kruskal–Wallis test, and pairwise comparisons were performed with the Conover test (kwAllPairsConoverTest function in PMCMRplus library, 1.9.12). A beta diversity analysis was performed using the Bray–Curtis dissimilarity index, calculated from microbial abundance data transformed into relative abundances at the genus level. The Principal Coordinate Analysis (PCoA) was visualized using the MicrobiotaProcess package, v. 1.18.0. To statistically assess differences between microbial communities across sampling points (phenophases) and treatment groups, a permutational multivariate analysis of variance (PERMANOVA) was conducted using the adonis2 function (vegan R package, v. 2.6-10). Permutations were constrained within plot IDs to account for paired samples. For each phenophase, pairwise comparisons among treatments were performed using the pairwise.adonis2 function (pairwiseAdonis package, v. 0.4.1). The top 50 genera, based on the higher abundance over all samples of soil, roots, seeds, or manure and phyla were presented with heatmaps using the complexheatmap package v. 2.22.0. If *Microbacterium* was not in the top 50 taxa, it was additionally appended as *Microbacterium* and also as *Micrococcales* and *Microbacteriaceae*. A differential abundance analysis was performed with ancombc2 (ancombc, v. 2.8.1) for each sampling point for soil, roots, and seeds with the following parameters: The genus was set for the taxa level, and the taxa with prevalence in less than 30% of the samples were removed. *p*-values were adjusted using the Benjamini–Hochberg (BH) method, with the significance level set to 0.1. Only taxa that passed the sensitivity analysis were considered differentially abundant between groups. To explore relationships between plant performance traits and root-associated bacterial communities, a redundancy analysis (RDA) was conducted using the vegan package in R. Prior to RDA, the genus-level abundance table was subjected to a Hellinger transformation to normalize the data. Plant and corn traits were used as explanatory variables. The contributions of bacterial genera to RDA axes were calculated, and the top 20 contributing genera were visualized using ggplot2, v. 3.5.1. In addition, Spearman’s rank correlation analysis was performed to assess the strength and direction of associations between individual bacterial genera and plant traits. For each genus–trait pair, *p*-values were adjusted for multiple testing using the BH method, with the significance level set to 0.1.

### 4.5. Bacterial Candidates’ Genome Sequencing

For whole-genome sequencing (WGS) of the bacterial candidates *Bacillus subtilis* sp. *subtilis* BM-15a and *Microbacterium* sp. AL-11a from the phytobiotic formulation, DNA samples, previously isolated with the ZymoBIOMICS^TM^ DNA Mini Kit D4300 (Zymo Research, Irvine, CA, USA), used according to the manufacturer’s instructions, were sent to MicrobesNG in Birmingham, England. The quality of the sequencing library was assessed using an Agilent Bioanalyzer 2100 (Agilent Technologies, Santa Clara, CA, USA) with the Agilent DNA 1000 kit (Agilent Technologies, Santa Clara, CA, USA). Sequencing was carried out on an Illumina MiSeq platform, utilizing 2 × 250 bp paired-end sequencing with the MiSeq Reagent Kit v3 (Illumina Inc., San Diego, USA), following the manufacturer’s instructions. Adapter removal and quality trimming were performed using Trim Galore! v0.6.6, a wrapper script for Cutadapt and FastQC. In addition to the default trimming parameters, 10 bases were removed from the 5′ end, and 2–4 bases were trimmed from the 3′ end of the reads. The additional trimming from the 3′ end helps eliminate low-quality bases that commonly accumulate toward the end of sequencing reads, improving overall sequence accuracy and downstream analysis reliability. Bacterial genome assembly was performed using Unicycler v0.4.8 with the “Illumina-only assembly” method [131], optimized for short-read sequencing data. The completeness and quality of the assembled genomes were assessed using BUSCO v5.2.2 (Benchmarking Universal Single-Copy Orthologs) [132]. The analysis was conducted with lineage-specific datasets, micrococcales_odb10 and bacillales_odb10, to evaluate the presence of conserved single-copy orthologs and to determine the completeness of the assemblies. Genome annotation was conducted using the DFAST web server [133] with default parameters. To verify taxonomic identity, the Taxonomy Check tool was used. Functional annotation was performed by scanning TIGRFAM protein families with HMM (Hidden Markov Model) and COG (Clusters of Orthologous Groups) using RPSBLAST (Reverse Position-Specific BLAST). Additionally, PFAM annotation was carried out using the Pfam 34 database and HMMER v3.3.2 [134]. The fIDBAC automated workflow was used for calculating the average nucleotide identity (ANI), which is a key metric for determining bacterial species relatedness based on whole-genome comparisons [135].

A phylogenetic analysis was performed with PhyloPhlAn 3.0.2. [136] with the following options: --trim greedy –not_variant_threshold 0.99 --remove_fragmentary_entries --fragmentary_threshold 0.67 --min_num_entries <total number of genomes> -t a --diversity low.

## 5. Conclusions

This study demonstrates that the combined application of poultry manure and seed-applied beneficial bacteria positively influences the maize bacteriobiome, enhancing microbial diversity, promoting ecological balance, and supporting plant development. While poultry manure enriched soil microbial communities in early stages, the seed coating with *B. subtilis* BM-15a and *Microbacterium* sp. AL-11a contributed to more balanced and resilient root-associated microbiota over time. Genomic insights revealed the inoculants’ multifunctional traits—*B. subtilis* BM-15a’s biocontrol and nutrient acquisition genes and the novel *Microbacterium* AL-11a’s stress adaptation mechanisms—supporting their complementary roles in plant growth. The synergy of these treatments promoted beneficial taxa associated with plant growth and health, while maintaining the stability of the indigenous soil microbiome. Despite some drawbacks observed in specific microbial groups, particularly in roots, the application of bio-based solutions, such as PHY and PM, represents a sustainable strategy for improving crop productivity and soil health, aligning with the goals of environmentally conscious agriculture. Future work should focus on optimizing delivery methods and ensuring long-term field efficacy, while deepening our understanding of microbial responses to validate ecological consequences. Importantly, these findings highlight the potential for developing tailored bacterial consortia that can be applied not only to maize but more broadly across diverse crops and agroecosystems—offering scalable, adaptable solutions for sustainable agriculture in variable environmental and soil health contexts.

## Figures and Tables

**Table 1:** Genome sequence features of *B. subtilis* sp. *subtilis* and related *B. subtilis* strains.

Strain	Source and Year of Isolation	Contigs (N)	N50 (bp)	Genome Size (bp)	GC Content (%)	Genes	Protein Coding Sequences (CDSs)	Reference
*Bacillus subtilis* sp. *subtilis* BM-15a	Soil, Serbia, 2019	73	447,052	3,955,365	43.9	4024	3956	This work
*Bacillus subtilis* sp. *subtilis* SSJ1 GCF_003665255.1	Cheonggukjang, Republic of Korea, 2016	1	4,200,000	4,200,000	43.5	4418	4187	[30]
*Bacillus subtilis* sp. *subtilis* OH 131.1 GCF_000706705.1	Wheat anther, USA, 2014	1	4,000,000	4,000,000	44	4171	4061	[31]
*Bacillus subtilis* sp. *subtilis* IITK SM1 GCF_003426125.1	Food waste compost, India, 2018	43	238,200	4,100,000	43.5	4255	4005	[32]

For both species, 99.5% genome completeness was obtained based on Busco results (0.2% out of 99.5% were associated with duplicated Busco genes).

**Table 2:** Comparative genomic analysis of *Bacillus subtilis* sp. *subtilis* assemblies based on average nucleotide identity (part of the table).

Related Genome	ANI	Matched	Fragments	Subspecies
BM-15a (our strain)	100	1305	1305	*Bacillus subtilis* sp. *subtilis*
GCF_000706705.1	99.4345	1276	1305	OH 131.1
GCF_003665255.1	99.3279	1281	1305	SSJ-1
GCF_003426125.1	98.9039	1282	1305	IITK SM1
GCF_003339745.1	98.8583	1245	1305	SBE1
GCF_000230755.1	98.8149	1272	1305	SC-8
GCF_001625135.1	98.1527	1252	1305	ALBA01
GCF_000349795.1	98.1265	1257	1305	BAB-1
GCF_000497365.1	98.0839	971	1305	MP11
GCF_003665215.1	98.0186	1160	1305	PJ-7
GCF_000227485.1	97.8605	1244	1305	RO-NN-1

**Table 3:** Genome sequence features of *Microbacterium* sp. and its related strains.

Strain	Source and Year of Isolation	Contigs (N)	N50 (bp)	Genome Size (bp)	GC Content (%)	Genes	Protein Coding Sequences (CDSs)	Reference
*Microbacterium* sp. AL-11a	Soil, Serbia, 2019	104	44,752	2,953,076	70.08	2748	2698	This work
*Microbacterium hydrothermale* (GCF_004854025.1)	Mirabilis jalapa, India, 2019	18	503,505	3,580,540	71	3379	3317	[33]
*Microbacterium testaceum* (GCF_006539145.1)	Chinese paddy, Japan, 2019	28	249,634	3,590,404	69.5	3402	3339	[34]
*Microbacterium enclense* (GCF_900096885.1)	N/A	15	302,128	3,666,504	70.5	3403	3322	[35]
*Microbacterium proteolyticum* (GCF_014192415.1)	N/A	10	1,816,927	3,482,219	70	3261	3198	[36]

**Table 4:** Comparative genomic analysis of *Microbacterium* sp. assemblies based on average nucleotide identity (part of the table).

Related Genome	ANI	Matched	Fragments	Species
AL-11a (our strain)	100	930	935	*Microbacterium* sp.
GCF_004854025.1	84.036	686	935	*Microbacterium hydrothermale*
GCF_900096885.1	83.698	680	935	*Microbacterium enclense*
GCF_006539145.1	83.276	649	935	*Microbacterium testaceum*
GCF_014192415.1	83.055	675	935	*Microbacterium proteolyticum*
GCF_014779795.1	80.743	482	935	*Microbacterium helvum*
GCF_000956465.1	80.501	478	935	*Microbacterium trichothecenolyticum*
GCF_008017415.1	80.496	524	935	*Microbacterium hatanonis*
GCF_019511665.1	80.466	503	935	*Microbacterium jejuense*
GCF_006783905.1	80.441	483	935	*Microbacterium kyungheense*
GCF_015278255.1	80.439	482	935	*Microbacterium hibisci*

## Data Availability

The original data presented in this study are openly available in NCBI at http://www.ncbi.nlm.nih.gov/bioproject/1246115 (accessed on 3 April 2025) or PRJNA1246115 under the accession numbers from SAMN47765350 to SAMN47765445.

## Supplementary Materials

The following supporting information can be downloaded at: https://www.mdpi.com/article/10.3390/plants14121753/s1. Table S1. Summary of sequence read processing for maize root, rhizosphere, seed and manure samples across different treatments and phenophases. The table includes the number of input reads, reads passing filtering, denoised and non-chimeric reads, along with the corresponding percentages of reads retained after each step. Table S2. Results of the differential abundance analysis. Taxa identified as statistically significantly differentially abundant compared to the control (uninoculated seed with no poultry manure (Un)) are marked with an asterix (*). Treatments include FitoBiotic Basic (*Phy*), poultry manure (*Pm*), and their combination (*Phy* + *Pm*). Table S3. Complete table of the comparative genomic analysis of *Bacillus subtilis* ssp. *subtilis* assemblies, based on average nucleotide identity. Table S4. Complete table of the comparative genomic analysis of *Microbacterium* sp. assemblies, based on average nucleotide identity. Table S5. Table of Spearman’s correlation assessing the strength and direction of associations between individual bacterial genera and plant traits. Figure S1. A heatmap of scaled average abundances of the 37 most prevalent bacterial phyla across all soil samples, including negative controls and treatments, throughout the four phenophases. Figure S2. A heatmap of scaled average abundances of the 34 most prevalent bacterial phyla across all root samples, including negative controls and treatments, throughout the three phenophases. Figure S3. A heatmap of scaled average abundances of the 21 most prevalent bacterial phyla across all seed samples, including negative controls and treatments before and after harvest. Figure S4. A heatmap of average abundances of 17 most prevalent taxa in manure samples.
