# Supplementary material for: The Influence of Bacterial Inoculants and a Biofertilizer on Maize Cultivation and the Associated Shift in Bacteriobiota During the Growing Season
Source: Plants (Basel). 2025 Jun 7;14(12):1753. doi: 10.3390/plants14121753 (PMC12196507; doi:10.3390/plants14121753)
Supplement: Supplementary file 1 [file plants-14-01753-s001.zip › plants-3625701-supplementary.pdf]

Supplementary Materials

The Influence of Bacterial Inoculants and a Biofertilizer on Maize Cultivation and the Associated Shift in Bacteriobiota During the Growing Season

Katarina Kruščić [1,#], Aleksandra Jelušić [2,#], Matjaž Hladnik [3], Tamara Janakiev [1], Jovana Anđelković [4], Dunja Bandelj [3] and Ivica Dimkić [1,*]

[1] University of Belgrade, Faculty of Biology, Studentski trg 16, 11158 Belgrade, Serbia; katarina.kruscic@bio.bg.ac.rs; tamara.janakiev@bio.bg.ac.rs; ivicad@bio.bg.ac.rs
[2] Department of Life Sciences, University of Belgrade – Institute for Multidisciplinary Research, Belgrade, Serbia; aleksandra.jelusic@imsi.rs
[3] University of Primorska, Faculty of Mathematics, Natural Sciences and Information Technologies (FAMNIT), Glagoljaška 8, Sl-6000 Koper, Slovenia; matjaz.hladnik@famnit.upr.si
[4] University of Niš, Faculty of Sciences and Mathematics, Višegradska 33, 18106 Niš, Serbia; jovana.andjelkovic@pmf.edu.rs
# The authors with equal contribution
* Correspondence: ivicad@bio.bg.ac.rs; Tel./Fax: +381-11-2638500

Table S1. Summary of sequence read processing for maize root, rhizosphere, seed and manure samples across different treatments and phenophases. The table includes the number of input reads, reads passing filtering, denoised and non-chimeric reads, along with the corresponding percentages of reads retained after each step.

sample-id	input	filtered	percentage of input passed filter	denoised	non-chimeric	percentage of input non-chimeric
IIIKT1_1	96637	77705	80.41	74990	73488	76.05
IIIKT1_2	107270	86401	80.55	84185	83566	77.9
IIIKT1_3	66808	52527	78.62	50714	50141	75.05
IIIKT2_1	52039	41612	79.96	39759	38822	74.6
IIIKT2_2	61572	49304	80.08	47353	46986	76.31
IIIKT2_3	100139	79202	79.09	77154	75191	75.09
IIIKT3_1	68244	55218	80.91	53256	51937	76.1
IIIKT3_2	60780	46483	76.48	45033	44432	73.1
IIIKT3_3	97939	79501	81.17	77267	74396	75.96
IIIKT4_1	76913	61095	79.43	59619	59178	76.94
IIIKT4_2	62878	50958	81.04	49226	48490	77.12
IIIKT4_3	51860	41327	79.69	39793	39471	76.11
IIIZT1_1	59023	44669	75.68	41736	41171	69.75
IIIZT1_2	47889	36085	75.35	33488	33037	68.99
IIIZT1_3	73717	56652	76.85	53280	52279	70.92
IIIZT2_1	76487	58689	76.73	54897	54030	70.64
IIIZT2_2	67162	51552	76.76	48513	47693	71.01
IIIZT2_3	52931	40244	76.03	37552	37167	70.22
IIIZT3_1	59024	45018	76.27	41887	41115	69.66
IIIZT3_2	60422	46121	76.33	43164	42621	70.54
IIIZT3_3	93720	73429	78.35	69513	67853	72.4
IIIZT4_1	76945	59844	77.78	56306	55319	71.89
IIIZT4_2	54283	41923	77.23	39352	38740	71.37
IIIZT4_3	96559	71956	74.52	68557	67128	69.52
IIKT1_1	69387	55238	79.61	54517	52265	75.32
IIKT1_2	62829	52501	83.56	51689	49786	79.24
IIKT1_3	109952	89742	81.62	88258	84930	77.24
IIKT2_1	61233	50008	81.67	48908	46391	75.76
IIKT2_2	96677	80513	83.28	79526	73548	76.08
IIKT2_3	82234	68917	83.81	68037	65002	79.05
IIKT3_1	138066	115973	84	114442	112009	81.13
IIKT3_2	74246	59671	80.37	58519	55215	74.37
IIKT3_3	61742	46414	75.17	45555	43716	70.8
IIKT4_1	111486	90122	80.84	88389	87449	78.44
IIKT4_2	71241	58863	82.63	57211	55686	78.17
IIKT4_3	44559	36595	82.13	35641	34911	78.35

IIZ2_1	104852	83871	79.99	80361	78026	74.42
IIZ2_2	51353	38454	74.88	35898	35261	68.66
IIZ2_3	71593	56552	78.99	53467	52412	73.21
IIZT1_1	52878	39848	75.36	37140	36498	69.02
IIZT1_2	57910	45113	77.9	42254	41591	71.82
IIZT1_3	57236	44606	77.93	41878	41104	71.81
IIZT3_1	66096	50861	76.95	47925	46993	71.1
IIZT3_2	77650	60071	77.36	56703	56030	72.16
IIZT3_3	91591	66419	72.52	63390	62573	68.32
IIZT4_1	83382	65710	78.81	62211	60948	73.09
IIZT4_2	67705	53111	78.44	49936	48578	71.75
IIZT4_3	78109	62902	80.53	59903	58240	74.56
IMT3_1	88739	76429	86.13	74637	62265	70.17
IMT3_2	81637	70110	85.88	69203	67688	82.91
IMT3_3	82566	72043	87.26	70020	55402	67.1
IST1_1	45201	21120	46.72	20907	20735	45.87
IST1_2	59855	51956	86.8	51908	51819	86.57
IST1_3	83274	42425	50.95	42179	41818	50.22
IST2_1	72849	48903	67.13	48645	48417	66.46
IST2_2	71056	42375	59.64	42234	41998	59.11
IST2_3	62900	39680	63.08	39500	39164	62.26
IVKT1_1	58554	48005	81.98	46423	45420	77.57
IVKT1_2	60150	47648	79.22	46175	45556	75.74
IVKT1_3	147631	118519	80.28	116351	115262	78.07
IVKT2_1	51086	40473	79.23	39126	38714	75.78
IVKT2_2	53158	42592	80.12	41074	40693	76.55
IVKT2_3	80584	60743	75.38	59071	57915	71.87
IVKT3_1	96801	78115	80.7	76260	75066	77.55
IVKT3_2	79877	64687	80.98	63101	62190	77.86
IVKT3_3	692393	553930	80	549980	535801	77.38
IVKT4_1	73683	58124	78.88	56507	55420	75.21
IVKT4_2	63681	50596	79.45	49148	48167	75.64
IVKT4_3	63807	51271	80.35	49774	48694	76.31
IVST1_1	88763	43321	48.81	43154	42976	48.42
IVST1_2	66807	43797	65.56	43667	43617	65.29
IVST1_3	89904	35586	39.58	35365	35203	39.16
IVST2_1	67017	37315	55.68	37126	36965	55.16
IVST2_2	81439	54111	66.44	53902	53715	65.96
IVST2_3	72260	49359	68.31	49155	48848	67.6
IVST3_1	66756	31898	47.78	31748	31305	46.89
IVST3_2	56652	30945	54.62	30824	30341	53.56
IVST3_3	83995	65529	78.02	65306	63344	75.41
IVST4_1	43299	23829	55.03	23686	23630	54.57
IVST4_2	47266	36130	76.44	36058	34773	73.57

IVST4_3	58761	33545	57.09	33401	33210	56.52
IVZKT2_1	56790	42167	74.25	39617	39234	69.09
IVZKT2_2	63111	48669	77.12	45768	45252	71.7
IVZKT2_3	69267	52831	76.27	49359	48518	70.04
IVZT1_1	63436	49651	78.27	46379	45585	71.86
IVZT1_2	55498	42153	75.95	39128	38666	69.67
IVZT1_3	105122	77787	74	73671	72170	68.65
IVZT3_1	61809	46868	75.83	43780	43099	69.73
IVZT3_2	46128	34822	75.49	32225	31763	68.86
IVZT3_3	89536	66882	74.7	62883	61690	68.9
IVZT4_1	78733	60021	76.23	56217	55148	70.04
IVZT4_2	84690	63725	75.25	60190	59341	70.07
IVZT4_3	60434	45187	74.77	42041	41521	68.7
IZT1_1	69477	52427	75.46	49589	48754	70.17
IZT1_2	69112	51564	74.61	48774	47917	69.33
IZT1_3	55858	42011	75.21	39140	38693	69.27

Table S2. Results of the differential abundance analysis. Taxa identified as statistically significantly differentially abundant compared to the control (uninoculated seed with no poultry manure (Un)) are marked with an asterix (*). Treatments include FitoBiotic Basic (*Phy*), poultry manure (*Pm*), and their combination (*Phy* + *Pm*).

Phenophase	Site	Taxon	Group	logFC
II	Root	*Cellulosimicrobium*	Phy - Un	-1.09
		Cellulosimicrobium	Pm - Un	-1.71*
		Cellulosimicrobium	Phy and Pm - Un	-1.70*
		Chryseobacterium	Phy - Un	0.23
		Chryseobacterium	Pm - Un	-0.32
		Chryseobacterium	Phy and Pm - Un	-2.38*
		Pedobacter	Phy - Un	-0.11
		Pedobacter	Pm - Un	-0.78
		Pedobacter	Phy and Pm - Un	-2.12*
		Phyllobacterium	Phy - Un	-0.58
		Phyllobacterium	Pm - Un	-1.70*
		Phyllobacterium	Phy and Pm - Un	-1.97*
		Sphingobacterium	Phy - Un	-0.47
		Sphingobacterium	Pm - Un	-0.83
		Sphingobacterium	Phy and Pm - Un	-3.14*
		Sphingopyxis	Phy - Un	-0.84
		Sphingopyxis	Pm - Un	0.24
		Sphingopyxis	Phy and Pm - Un	-2.19*
		Stenotrophomonas	Phy - Un	0.39
		Stenotrophomonas	Pm - Un	-0.34
		Stenotrophomonas	Phy and Pm - Un	-2.48*
III	Soil	*11-24*	Phy - Un	0.08
		11-24	Pm - Un	1.4*
		11-24	Phy and Pm - Un	0.01
IV	Soil	*Ellin6055*	Phy - Un	-0.61
		Ellin6055	Pm - Un	-0.63*
		Ellin6055	Phy and Pm - Un	-6.01
		Shimazuella	Phy - Un	-0.13
		Shimazuella	Pm - Un	-0,73*
		Shimazuella	Phy and Pm - Un	-0.18
	Root	*Flavisolibacter*	Phy - Un	0.51
		Flavisolibacter	Pm - Un	0.86*
		Flavisolibacter	Phy and Pm - Un	0.42
		Stenotrophomonas	Phy - Un	-1.71
		Stenotrophomonas	Pm - Un	-1.59
		Stenotrophomonas	Phy and Pm - Un	-2.11*

Table S3. Complete table of the comparative genomic analysis of *Bacillus subtilis* ssp. *subtilis* assemblies, based on average nucleotide identity.

Related genome	ANI	Matched	Fragments	Subspecies
our strain	100	1305	1305	our strain
GCF_000706705.1	99.4345	1276	1305	OH 131.1
GCF_003665255.1	99.3279	1281	1305	SSJ-1
GCF_003426125.1	98.9039	1282	1305	IITK SM1
GCF_003339745.1	98.8583	1245	1305	SBE1
GCF_000230755.1	98.8149	1272	1305	SC-8
GCF_000321395.1	98.8094	1267	1305	BSP1
GCF_012648205.1	98.7813	1278	1305	UCMB5121
GCF_012648345.1	98.78	1280	1305	UCMB5021
GCF_000740485.1	98.7787	1267	1305	NDfood
GCF_000699465.1	98.7768	1252	1305	JH642 substr. AG174
GCF_002202035.1	98.7684	1267	1305	SRCM101392
GCF_012931705.1	98.7673	1252	1305	SAMN14645746
GCF_000740475.1	98.7654	1270	1305	NDmed
GCF_001660525.1	98.7614	1220	1305	delta6
GCF_000699525.1	98.761	1258	1305	AG1839
GCF_014389245.2	98.7468	1268	1305	DSM 10
GCF_001703495.1	98.7452	1269	1305	168G
GCF_001697265.1	98.745	1271	1305	KCTC 3135
GCF_000696635.1	98.7419	1271	1305	168
GCF_012030675.1	98.7407	1263	1305	SMY
GCF_000186085.1	98.7323	1270	1305	NCIB 3610
GCF_003336895.2	98.7308	1257	1305	OGU1
GCF_000155325.1	98.7299	1270	1305	168
GCF_000155375.1	98.7272	1268	1305	SMY
GCF_013332455.1	98.7264	1273	1305	GR2.1
GCF_006088795.1	98.7243	1273	1305	NCIB 3610
GCF_002009135.1	98.7207	1269	1305	168
GCF_006741845.1	98.7183	1271	1305	NBRC 13719
GCF_012931705.2	98.7181	1260	1305	168
GCF_013867545.1	98.7176	1272	1305	s-2
GCF_002009095.1	98.7167	1269	1305	168
GCF_003336895.1	98.7132	1258	1305	SAMN09217786
GCF_002998755.1	98.7085	1259	1305	SYST2
GCF_009741365.1	98.7059	1262	1305	PY79
GCF_000827065.1	98.7056	1275	1305	3NA
GCF_000344745.1	98.7045	1275	1305	6051-HGW
GCF_000155355.1	98.7045	1265	1305	JH642
GCF_000789275.1	98.7043	1275	1305	168
GCF_000009045.1	98.7043	1275	1305	168

GCF_014389245.1	98.704	1276	1305	SAMN15904628
GCF_000245295.1	98.6993	1248	1305	AUSI98
GCF_001750745.1	98.6979	1275	1305	QB5412
GCF_002998795.1	98.6973	1264	1305	NMSX4
GCF_013009385.1	98.6908	1265	1305	168
GCF_011800745.1	98.6886	1260	1305	EA-CB0575
GCF_001750765.1	98.6786	1016	1305	QB5413
GCF_010094155.1	98.6643	1260	1305	ABP2
GCF_004328925.1	98.6171	1278	1305	G7
GCF_001541905.1	98.6012	1277	1305	CU1050
GCF_002173695.1	98.4753	1205	1305	SRCM101444
GCF_002201955.1	98.4651	1207	1305	SRCM100761
GCF_007666785.1	98.4631	1175	1305	DE0515
GCF_002173715.1	98.4592	1209	1305	SRCM100757
GCF_007666815.1	98.453	1174	1305	DE0514
GCF_018454405.1	98.4489	1269	1305	A1 - Midalam
GCF_014298115.1	98.4455	1251	1305	ONU 559
GCF_003931545.1	98.4334	1255	1305	BE27
GCF_002142515.1	98.4116	1181	1305	M1
GCF_001661555.1	98.4114	1247	1305	Y3
GCF_001057075.1	98.4038	1245	1305	516_BAMY
GCF_002865705.1	98.3888	1213	1305	BSP2
GCF_009996815.1	98.3472	1262	1305	A52
GCF_000497345.1	98.3309	1101	1305	MP9
GCF_002865715.1	98.317	1220	1305	BSP4
GCF_003665315.1	98.3049	1177	1305	N2-2
GCF_003665355.1	98.3029	1175	1305	N3-1
GCF_003665295.1	98.2957	1178	1305	N4-2
GCF_003665335.1	98.2695	1178	1305	N1-1
GCF_014961985.1	98.2596	1175	1305	CMIN-4
GCF_000828495.1	98.2541	1172	1305	B4067
GCF_018986855.1	98.2534	1183	1305	Miz-8
GCF_003665195.1	98.2351	1189	1305	GFR-12
GCF_003665275.1	98.2317	1190	1305	2RL2-3
GCF_003665395.1	98.2281	1190	1305	2KL1
GCF_002906075.1	98.222	1177	1305	SRCM101384
GCF_002201995.1	98.22	1182	1305	SRCM100333
GCF_002173615.1	98.1983	1186	1305	SRCM101441
GCF_003665235.1	98.1904	1187	1305	MH-1
GCF_001465815.1	98.1555	1253	1305	BSD-2
GCF_001625135.1	98.1527	1252	1305	ALBA01
GCF_000349795.1	98.1265	1257	1305	BAB-1
GCF_000497365.1	98.0839	971	1305	MP11
GCF_003665215.1	98.0186	1160	1305	PJ-7

GCF_000227485.1	97.8605	1244	1305	RO-NN-1

Table S4. Complete table of the comparative genomic analysis of *Microbacterium* sp. assemblies, based on average nucleotide identity.

Related genome	ANI	Matched	Fragments	Species
our strain	100	930	935	our strain
GCF_004854025.1	84.0355	686	935	*Microbacterium hydrothermale*
GCF_900096885.1	83.6978	680	935	*Microbacterium enclense*
GCF_006539145.1	83.2762	649	935	*Microbacterium testaceum*
GCF_014192415.1	83.0546	675	935	*Microbacterium proteolyticum*
GCF_014779795.1	80.7429	482	935	*Microbacterium helvum*
GCF_000956575.1	80.6138	471	935	*Microbacterium ketosireducens*
GCF_014208045.1	80.5912	468	935	*Microbacterium thalassium*
GCF_004564355.1	80.5375	485	935	*Microbacterium wangchenii*
GCF_018588945.1	80.517	445	935	*Microbacterium flavescens*
GCF_017831975.1	80.5035	482	935	*Microbacterium terrae*
GCF_000956465.1	80.5006	478	935	*Microbacterium trichothecenolyticum*
GCF_008017415.1	80.4964	524	935	*Microbacterium hatanonis*
GCF_019511665.1	80.466	503	935	*Microbacterium jejuense*
GCF_006783905.1	80.4412	483	935	*Microbacterium kyungheense*
GCF_015278255.1	80.439	482	935	*Microbacterium hibisci*
GCF_003991875.1	80.3726	501	935	*Microbacterium lemovicicum*
GCF_001974985.1	80.3578	445	935	*Microbacterium aurum*
GCF_017876655.1	80.3459	486	935	*Microbacterium imperiale*
GCF_003651225.1	80.3404	466	935	*Microbacterium telephonicum*
GCF_006716815.1	80.3328	450	935	*Microbacterium lacticum*
GCF_900292075.1	80.3242	454	935	*Microbacterium timonense*
GCF_008727775.1	80.2956	487	935	*Microbacterium lushaniae*
GCF_015278355.1	80.2881	465	935	*Microbacterium yannicii*
GCF_015278315.1	80.2817	491	935	*Microbacterium ureisolvens*
GCF_013409785.1	80.2657	469	935	*Microbacterium immunditiarum*
GCF_000956535.1	80.1753	409	935	*Microbacterium ginsengisoli*
GCF_007992455.1	80.1427	443	935	*Microbacterium saccharophilum*
GCF_014725695.1	80.1309	469	935	*Microbacterium hominis*
GCF_011759705.1	80.1115	461	935	*Microbacterium ulmi*
GCF_008710705.1	80.0834	460	935	*Microbacterium radiodurans*
GCF_001662775.1	80.0615	485	935	*Microbacterium arborescens*

GCF_016907295.1	80.0015	426	935	*Microbacterium dextranolyticum*
GCF_015278285.1	79.9165	412	935	*Microbacterium invictum*
GCF_007988825.1	79.8661	358	935	*Microbacterium aerolatum*
GCF_009735645.1	79.8467	396	935	*Microbacterium oryzae*
GCF_014204835.1	79.8444	415	935	*Microbacterium marinum*
GCF_018717645.1	79.8399	433	935	*Microbacterium flavum*
GCF_001652465.1	79.8265	450	935	*Microbacterium chocolatum*
GCF_900100885.1	79.8178	420	935	*Microbacterium pygmaeum*
GCF_900105335.1	79.7973	386	935	*Microbacterium paraoxydans*
GCF_015235415.1	79.7398	390	935	*Microbacterium paludicola*
GCF_000802305.1	79.7343	426	935	*Microbacterium mangrovi*
GCF_016907315.1	79.7174	366	935	*Microbacterium esteraromaticum*
GCF_014205075.1	79.7096	352	935	*Microbacterium ginsengiterrae*
GCF_016907555.1	79.7077	425	935	*Microbacterium laevaniformans*
GCF_013409745.1	79.6926	355	935	*Microbacterium pseudoresistens*
GCF_008868005.1	79.6678	399	935	*Microbacterium algeriense*
GCF_001552455.1	79.6469	375	935	*Microbacterium hydrocarbonoxydans NBRC 103074*
GCF_015278225.1	79.6277	488	935	*Microbacterium aquimaris*
GCF_000422405.1	79.6272	417	935	*Microbacterium luticocti DSM 19459*
GCF_004564075.1	79.6228	391	935	*Microbacterium sediminis*
GCF_900114965.1	79.6161	414	935	*Microbacterium azadirachtae*
GCF_008727755.1	79.6143	404	935	*Microbacterium caowuchunii*
GCF_019753765.1	79.5585	402	935	*Microbacterium marinilacus*
GCF_019308305.1	79.5567	369	935	*Microbacterium resistens*
GCF_014643695.1	79.5252	362	935	*Microbacterium album*
GCF_017876435.1	79.5248	417	935	*Microbacterium phyllosphaerae*
GCF_006539765.1	79.4897	381	935	*Microbacterium liquefaciens*
GCF_016907255.1	79.4293	336	935	*Microbacterium keratanolyticum*
GCF_001975955.2	79.3654	418	935	*Microbacterium oleivorans*
GCF_003367705.1	79.3219	404	935	*Microbacterium foliorum*
GCF_002872075.1	79.3215	383	935	*Microbacterium kitamiense*
GCF_002812805.1	79.2987	384	935	*Microbacterium lacus*
GCF_015565955.1	79.2851	355	935	*Microbacterium schleiferi*
GCF_003991855.1	79.2794	368	935	*Microbacterium oxydans*
GCF_008710745.1	79.2774	441	935	*Microbacterium rhizomatis*

GCF_000455825.1	79.2165	365	935	*Microbacterium maritypicum MF109*
GCF_014635185.1	79.2049	361	935	*Microbacterium murale*
GCF_004135285.1	79.1585	346	935	*Microbacterium protaetiae*
GCF_000422385.1	79.122	342	935	*Microbacterium indicum DSM 19969*
GCF_006716345.1	79.1216	378	935	*Microbacterium saperdae*
GCF_000826185.2	79.1182	335	935	*Microbacterium gorillae*
GCF_000763375.1	79.0549	364	935	*Microbacterium profundi*
GCF_002970955.1	78.8131	347	935	*Microbacterium halophytorum*
GCF_003327285.1	78.8053	328	935	*Microbacterium sorbitolivorans*
GCF_011326725.1	78.7784	336	935	*Microbacterium excoecariae*
GCF_009745985.1	78.7453	377	935	*Microbacterium karelineae*
GCF_000422745.1	78.6168	322	935	*Microbacterium gubbeenense DSM 15944*
GCF_003254645.1	78.6	316	935	*Microbacterium suaedae*
GCF_014646015.1	78.5652	304	935	*Microbacterium nanhaiense*
GCF_003569805.1	78.5136	299	935	*Microbacterium halotolerans*
GCF_900105715.1	78.2702	270	935	*Microbacterium humi*
GCF_014640975.1	78.1903	288	935	*Microbacterium faecale*
GCF_008017445.1	78.1413	195	935	*Microbacterium mitrae*
GCF_011046975.1	77.9161	176	935	*Microbacterium amylolyticum*
GCF_011047135.1	77.8074	213	935	*Microbacterium endophyticum*
GCF_011761265.1	77.7895	208	935	*Microbacterium halimionae*

Table S5. Table of Spearman's correlation assessing the strength and direction of associations between individual bacterial genera and plant traits.

Site	Genera	Trait	rho	pvalue	p_adj
Soil	EPR3968-O8a-Bc78	Ustilago	-0,7990	0,0018	0,0775
	Hungateiclostridiaceae	Broken_plants	0,7964	0,0019	0,0775
	Dinghuibacter	Grain_yield	-0,8665	0,0003	0,0319
Root	*Acidibacter*	Plant_vigor	0,8362	0,0007	0,0420
	Comamonadaceae	Grain_moisture	0,7881	0,0023	0,0931
	Bradyrhizobium	Plant_vigor	0,8362	0,0007	0,0420

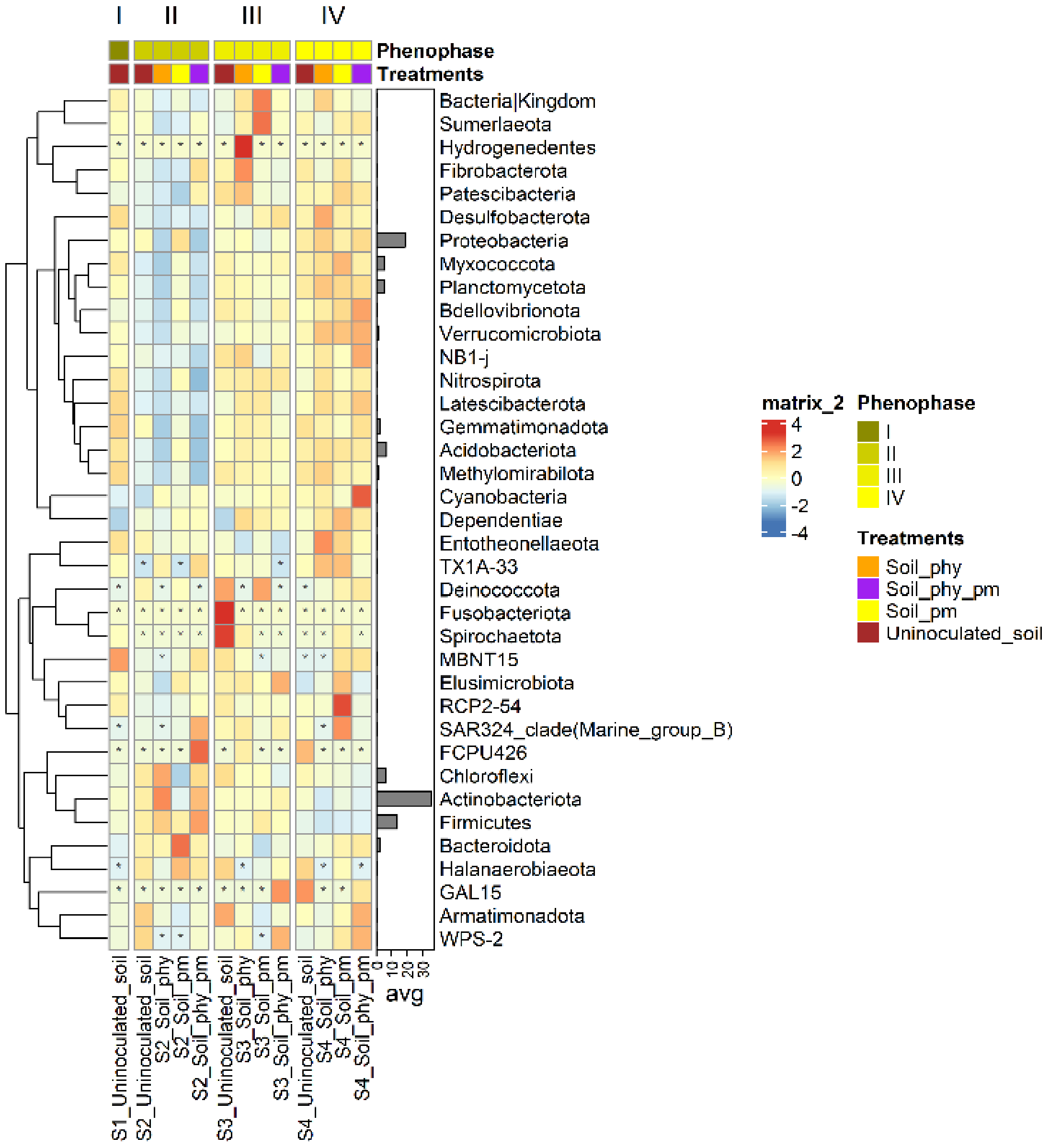

Figure S1. A heatmap of scaled average abundances of the 37 most prevalent bacterial phyla across all soil samples, including negative controls and treatments, throughout the four phenophases.

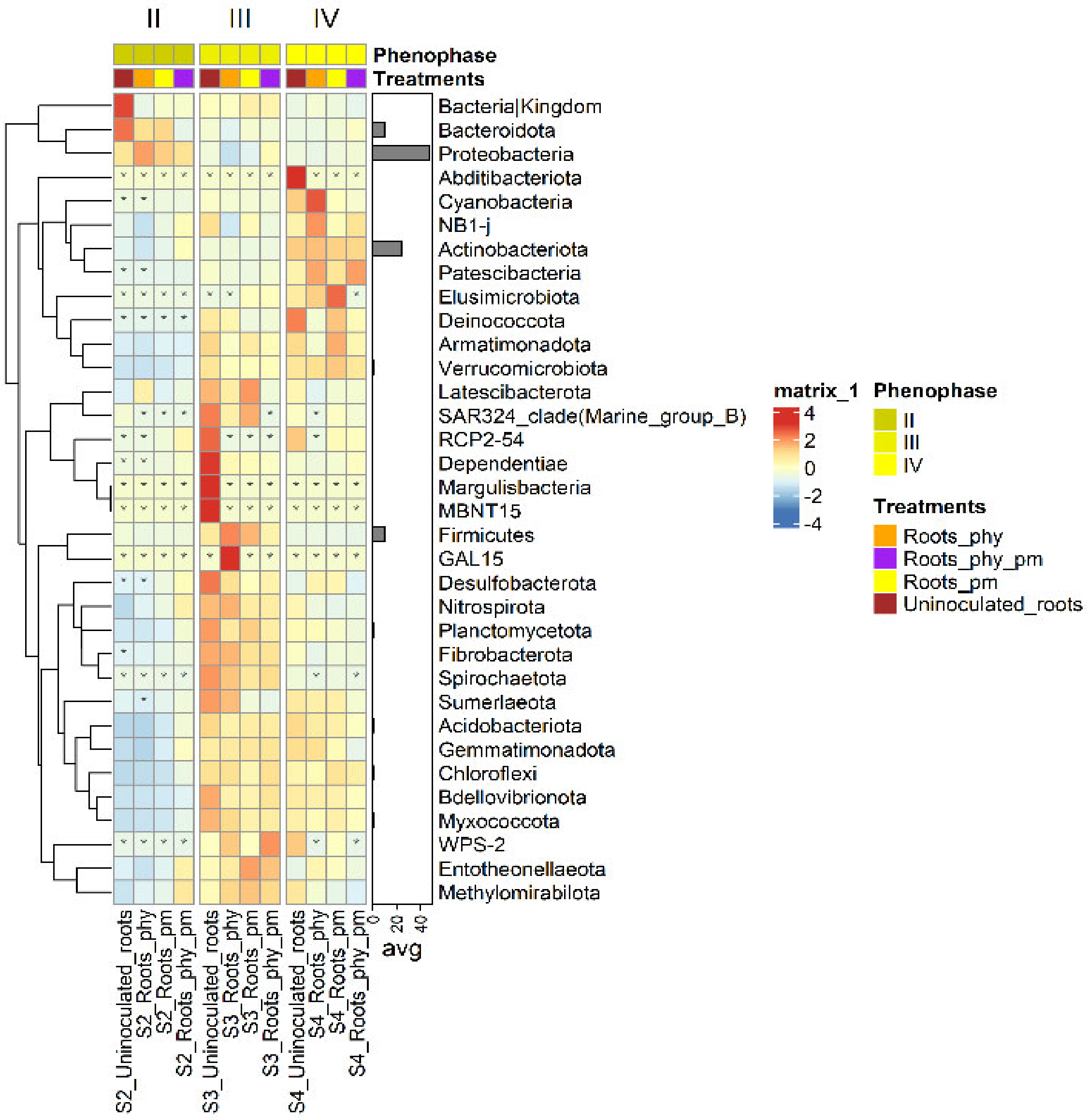

Figure S2. A heatmap of scaled average abundances of the 34 most prevalent bacterial phyla across all root samples, including negative controls and treatments, throughout the three phenophases.

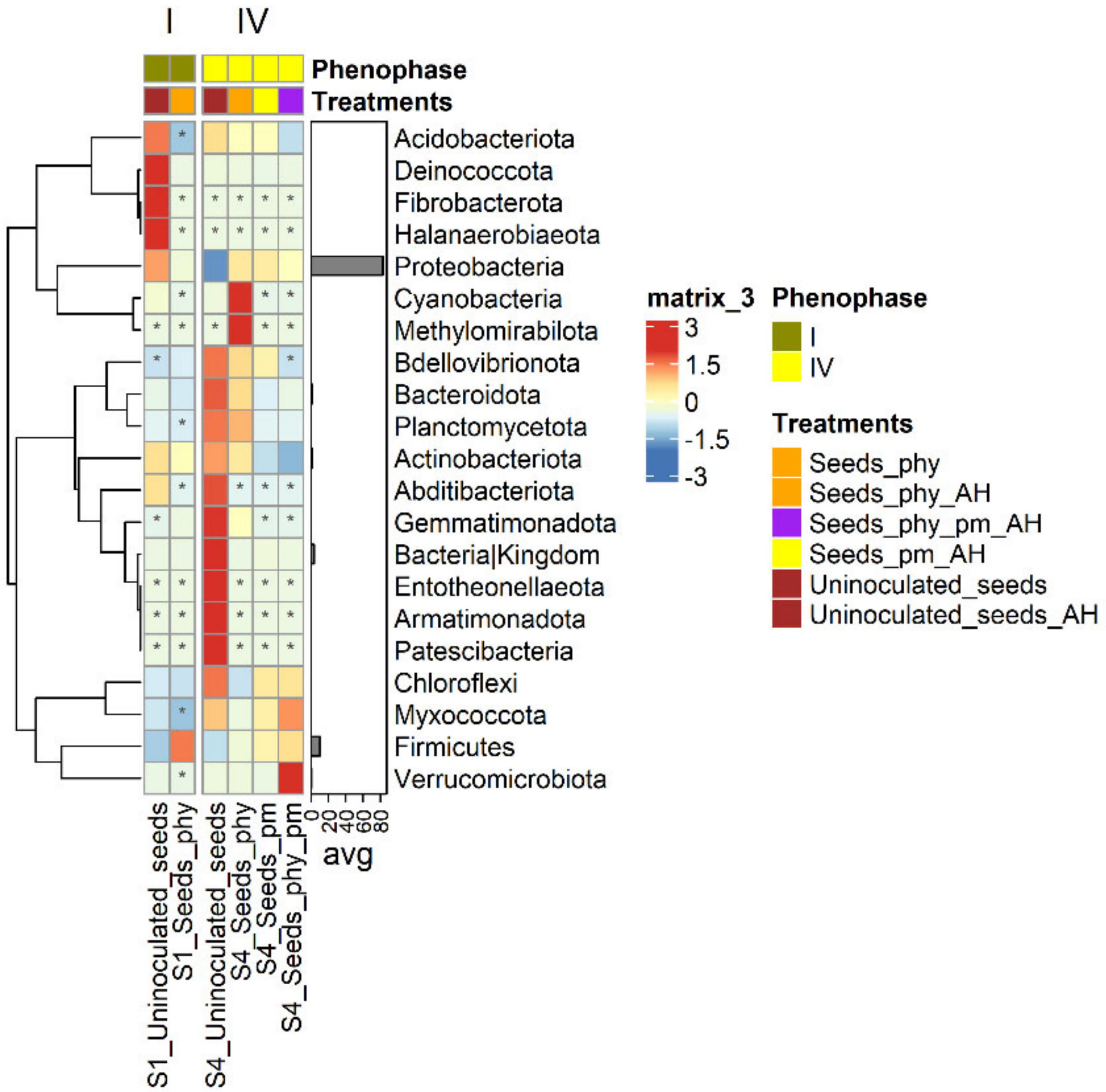

Figure S3. A heatmap of scaled average abundances of the 21 most prevalent bacterial phyla across all seed samples, including negative controls and treatments before and after harvest.

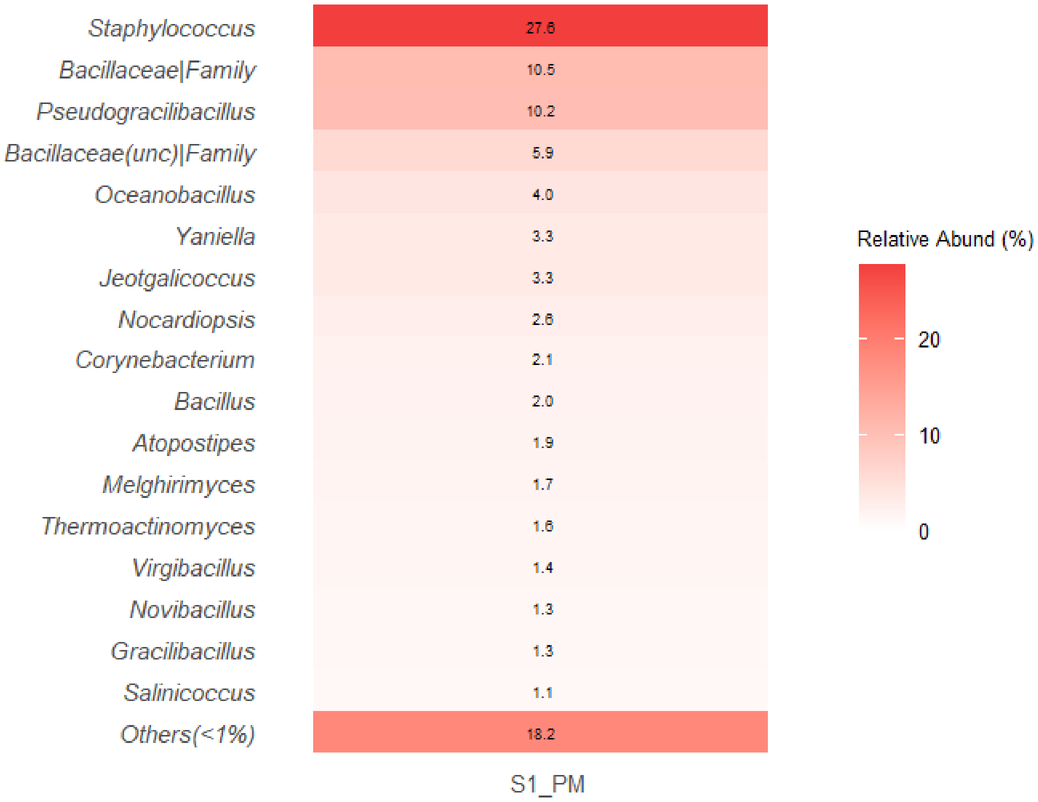

Figure S4. A heatmap of average abundances of 17 most prevalent taxa in manure samples.
